# Preoperative Creatinine Clearance and Mortality of Elective Cardiac Surgery in Hospitalization: A Secondary Analysis

**DOI:** 10.3389/fcvm.2021.712229

**Published:** 2022-01-27

**Authors:** Lu Chen, Yan He, Kai Song, Bingqian Zhang, Lin Liu

**Affiliations:** ^1^Department of Clinical Trials Centre, The Affiliated Hospital of Guizhou Medical University, Guiyang, China; ^2^The Affiliated Hospital of Guizhou Medical University, Guiyang, China

**Keywords:** creatinine clearance, elective cardiac surgery, mortality, in hospitalization, cohort study

## Abstract

**Objective:**

It has been reported that poor renal function before surgery is related to poor prognosis. However, there is no specific discussion on the ideal value of preoperative creatinine clearance. Consequently, our primary goal is to explore the correlation between baseline creatinine clearance and short-term mortality after cardiac surgery.

**Methods:**

We conducted a secondary data analysis based on a French cardiac surgery cohort. The cohort included 6,889 participants in a Paris university hospital from December 2005 to December 2012. The exposure variable and outcome variable used in this secondary analysis were the preoperative creatinine clearance rate and postoperative hospital mortality. Multivariate logistic regression and generalized additive models were employed.

**Results:**

The nonlinear relationship between the preoperative creatinine clearance rate and postoperative death was observed in this study. The preoperative creatinine clearance rate was negatively correlated with postoperative mortality in the range of 8.9–78.5 in patients younger than 80 years old (odds ratio = 0.98, 95% confidence interval 0.97–0.98, in Cockcroft Gault formulae). However, this effect characteristics reaches saturation after the preoperative creatinine clearance rate exceeds 78.5 (odds ratio = 0.99, 95% confidence interval 0.98–1.00, CG). In patients with history of thromboembolic event and coronary artery disease, the saturation effect were 30.8 mL.min^−1^ (CG) and 56.6 mL.min^−1^(CG).

**Conclusion:**

In the range of 8.9–78.5 (Cockcroft), an increase in preoperative creatinine clearance is associated with a decrease in postoperative mortality with patients younger than 80 years old. In patients with a history of embolism and coronary artery disease, the cut-off points of the reduction in preoperative creatinine clearance associated with a increase in postoperative mortality are 30.8 mL.min^−1^ and 56.6 mL.min^−1^.

## Introduction

The creatinine clearance rate (Ccr) is a common method of evaluating renal function. Compared with serum creatinine, creatinine clearance is a better indicator of renal function in risk stratification of patients undergoing cardiac surgery (1). The Cockcroft Gault (CG) and the Modification of Diet in Renal Disease (MDRD) formulae are the main formulae used to calculate Ccr (2, 3). Luciani, R et al. indicated that the CG Gault formula could be more powerful than the MDRD formula in preoperative prediction of early postoperative clinical outcomes in cardiac surgery in patients not affected by renal failure (4). Dardashti, A et al. used the MDRD formula because it has been widely used in studies in cardiac surgery and seems to be the most robust method when predicting outcome. They found that the poorest renal function was the best predictor of outcome (5).

Cardiac surgery is a higher risk operation. A study by O. Papachristofi found that 110,769 patients undergoing cardiac surgery had a hospital mortality rate of 3.1% (6). Patient risk accounted for 95.75% of the variation in in-hospital mortality seen in the study (7). Therefore, finding the risk factors related to mortality associated with cardiac surgery can help to stratify patients. Previous studies have reported that preoperative renal dysfunction is significantly and independently associated with mortality after cardiac surgery (8–10). However, limited by methodology and samples, studies with larger sample sizes are needed to clarify the true relationship between preoperative Ccr and postoperative in-hospital mortality. Therefore, we conducted a secondary analysis of Ccr and the risk of death after elective cardiac surgery based on a large-sample cardiac surgery cohort in France.

## Methods

### Patients and Data

This is a secondary analysis based on Jérôme Allyn's database (11). The patients underwent cardiac surgery with cardiopulmonary bypass at a University hospital between December 2005 and December 2012. In the original text, the author has stated that the research has been approved by the ethics committee (Institutional Review Board 00006477, Paris 7 University, AP-HP). As the original author stated, preoperative care of all patients was standardized, including anesthesia, monitoring techniques and normothermic cardiopulmonary bypass.

**Figure 1:**
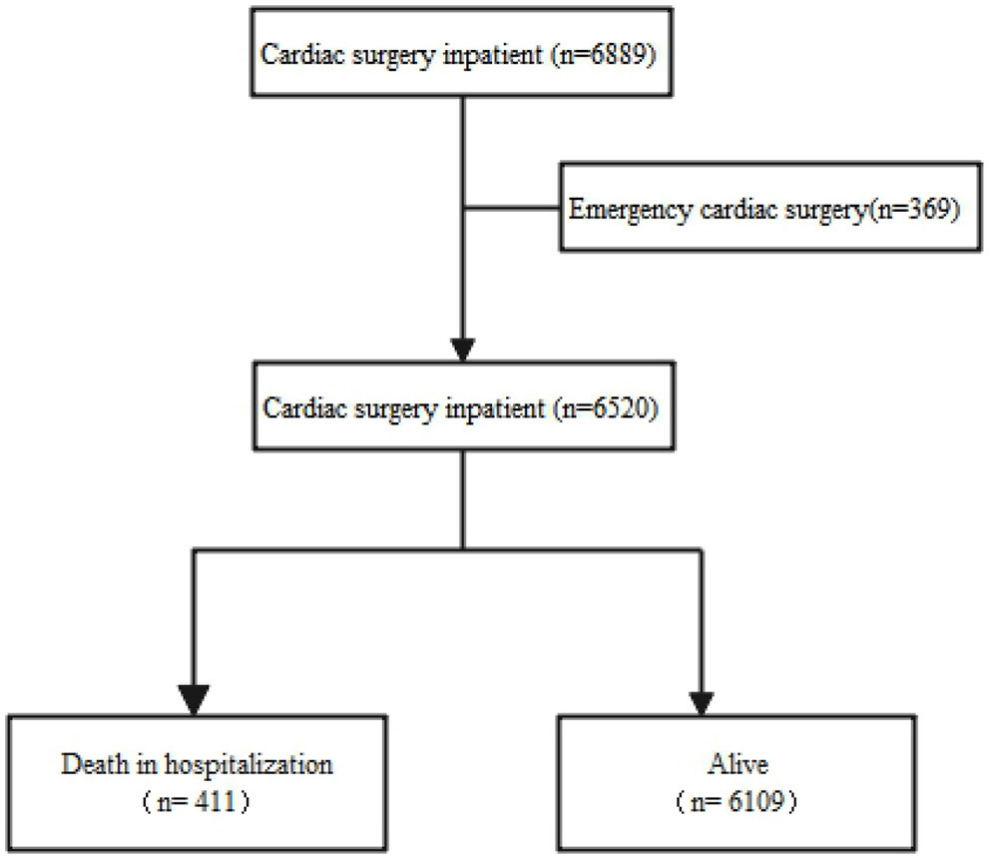
Flowchart.

In the present study, a total of 6,889 patients who underwent cardiac surgery were enrolled. After excluding 369 patients with emergency cardiac surgery, 6,520 patients were included in the data analysis (Figure 1). We examined the records on short-term death during hospitalization. The independent variable was creatinine clearance, and all patients were divided into four groups based on the classification of creatinine clearance rate: Ccr <30 mL.min^−1^ (severe damage); Ccr ≥30 mL.min^−1^, <60 mL.min^−1^ (moderate damage); Ccr ≥60 mL.min^−1^, <80 mL.min^−1^ (mild damage); and Ccr ≥80 mL.min^−1^ (normal renal function). Covariates involved in the secondary analysis were based on our clinical experience and previous literature (12, 13). Continuous variables included age, BMI and left ventricular ejection fraction. Categorical variables included sex, smoking status, poor mobility, comorbidities (hypertension, diabetes mellitus, dyslipidaemia, myocardial infarction <90 days, history of coronary artery disease, history of cardiac congestive failure, history of thromboembolic event, peripheral vascular disease, valve disease, ischemic stroke, chronic pulmonary disease, immunodeficiency, chronic kidney disease requiring dialysis, and New York Heart Association class (1, 2, 3 or 4), preoperative treatment (beta blockers, statins, calcium channel blockers, and angiotensin converting enzyme inhibitors) and number of diseased vessels (0, 1, 2, 3 or 4).

### Statistical Analysis

Consecutive variables are expressed as medians (P25, P75); categorical variables are reported as numbers (percentages). The baseline information was divided into four groups according to the clinical stage of Ccr after elective cardiac surgery during hospitalization. We conducted the analysis on raw data without interpolation because the proportion of missing variables was <1%. The “*P* value” and “*P* value^*^” shown in Table 1 refer to the results of the parametric test and the results of the nonparametric test, respectively. To investigate whether preoperative Ccr during hospitalization is correlated with mortality after elective cardiac surgery, we conducted two main steps to complete our statistical analysis. In the first step, weighted univariate and multivariate binary logistic regression were employed. We constructed three models: Model 1, no covariates were adjusted; Model 2, only adjusted for age and sex data; and Model 3, Model 2 with other covariates. These models are presented in Table 1. In the second step, to address for the nonlinearity of Ccr and mortality, a weighted generalized additive model (GAM) and smooth curve fitting (penalized spline method) were conducted. If nonlinearity was detected, we first calculated the inflection point using a recursive algorithm and then constructed a weighted two-piecewise linear regression model on both sides of the inflection point (14). We determined the best fit model (binary logistic regression model vs. two-piecewise linear regression model) based on the *P* values for the log likelihood ratio test. To ensure the robustness of the data analysis, we performed sensitivity analysis. First, we converted the Ccr into a categorical variable by clinical stage and calculated the P for trend to verify the results of Ccr as a continuous variable and to observe the possibility of nonlinearity. Second, we used the same adjustment strategy to analyse the Ccr calculated by the MDRD formula and the CG formula. All analyses were performed using the statistical software package R (http://www.R-project.org, The R Foundation) and EmpowerStats (http://www.Empowerstats.com, X&Y Solutions, Inc., Boston, MA). *P* < 0.05 (two-sided) were considered statistically significant.

**Table 1:** Preoperative characteristics and outcome of the entire study cohort based on Clinical staging of Ccr.

**Creatinine clearance (CG), mL.min^**−1**^**	**Missing data**	**Total (6,520)**	** <30 (274)**	**≥30, <60 (1,776)**	**≥60, <80 (1,641)**	**≥80 (2,792)**	***P*-value**	*P*-value^*^
Outcome (death)	0	411 (6.30%)	58 (21.17%)	165 (9.29%)	85 (5.18%)	97 (3.47%)	<0.001	-
**General description**								
Age, Median (Q1-Q3), years	0	65.00 (55.00–74.00)	75.00 (65.25–81.00)	75.00 (69.00–80.00)	68.00 (61.00–74.00)	56.00 (47.00–64.00)	<0.001	<0.001
BMI, Median (Q1-Q3), kg. m^−2^	19	26.03 (23.46–29.07)	23.57 (21.07–27.34)	24.68 (22.51–27.55)	25.95 (23.67–28.69)	27.17 (24.38–30.49)	<0.001	<0.001
Male, *n* (%)	0	4,449 (68.24%)	132 (48.18%)	996 (56.08%)	1,098 (66.91%)	2,198 (78.72%)	<0.001	–
Poor mobility	0	179 (2.75%)	17 (6.20%)	57 (3.21%)	49 (2.99%)	56 (2.01%)	<0.001	-
Smoking status (current/former smoker)	25	2,327 (35.83%) 1,009 (15.54%)	82 (30.15%) 17 (6.25%)	608 (34.41%) 139 (7.87%)	659 (40.31%) 185 (11.31%)	970 (34.82%) 660 (23.69%)	<0.001	–
Left ventricular ejection fraction, Median (Q1-Q3), %	2	60.00 (50.00–66.00)	55.00 (45.25–63.00)	60.00 (50.00–66.00)	60.00 (50.00–66.00)	60.00 (53.00–67.00)	<0.001	<0.001
New York Heart Association class1234	0	1,374 (21.07%) 603 (9.25%) 2,614 (40.09%) 1,929 (29.59%)	29 (10.58%) 9 (3.28%) 87 (31.75%) 149 (54.38%)	295 (16.61%) 98 (5.52%) 678 (38.18%) 705 (39.70%)	335 (20.41%) 150 (9.14%) 683 (41.62%) 473 (28.82%)	703 (25.18%) 342 (12.25%) 1,159 (41.51%) 588 (21.06%)	<0.001	–
**Comorbidities**, ***n*** **(%)**								
hypertension	0	3,678 (56.41%)	206 (75.18%)	1,171 (65.93%)	969 (59.05%)	1,311 (46.96%)	<0.001	–
Diabetes mellitus	2	1,674 (25.68%)	93 (33.94%)	459 (25.84%)	402 (24.51%)	715 (25.62%)	0.012	–
Dyslipidemia	0	3,288 (50.43%)	142 (51.82%)	926 (52.14%)	868 (52.89%)	1,338 (47.92%)	0.004	–
Myocardial infarction <90 days	0	440 (6.75%)	17 (6.20%)	106 (5.97%)	110 (6.70%)	204 (7.31%)	0.357	–
History of coronary artery disease	0	2,496 (38.28%)	84 (30.66%)	637 (35.87%)	658 (40.10%)	1,102 (39.47%)	0.002	–
History of cardiac congestive failure	0	1,047 (16.06%)	114 (41.61%)	383 (21.57%)	255 (15.54%)	289 (10.35%)	<0.001	–
History of thromboembolic event	0	353 (5.41%)	23 (8.39%)	110 (6.19%)	94 (5.73%)	125 (4.48%)	0.008	–
Peripheral vascular disease	0	905 (13.88%)	75 (27.37%)	301 (16.95%)	236 (14.38%)	285 (10.21%)	<0.001	–
Valve disease	0	3,810 (58.44%)	200 (72.99%)	1,211 (68.19%)	934 (56.92%)	1,449 (51.90%)	<0.001	–
Ischemic stroke	0	464 (7.12%)	36 (13.14%)	141 (7.94%)	128 (7.80%)	153 (5.48%)	<0.001	–
Chronic pulmonary disease	0	375 (5.75%)	19 (6.93%)	135 (7.60%)	80 (4.88%)	138 (4.94%)	<0.001	–
Chronic kidney disease requiring dialysis	0	57 (0.87%)	3 (1.09%)	17 (0.96%)	17 (1.04%)	20 (0.72%)	0.663	–
Immunodeficiency	0	88 (1.35%)	11 (4.01%)	18 (1.01%)	25 (1.52%)	34 (1.22%)	<0.001	–
Number of vessel-disease	0	3,211 (49.25%) 487 (7.47%) 624 (9.57%) 2,079 (31.89%) 119 (1.83%)	133 (48.54%) 33 (12.04%) 33 (12.04%) 69 (25.18%) 6 (2.19%)	874 (49.21%) 169 (9.52%) 172 (9.68%) 530 (29.84%) 31 (1.75%)	768 (46.80%) 138 (8.41%) 173 (10.54%) 529 (32.24%) 33 (2.01%)	1,415 (50.68%) 146 (5.23%) 245 (8.78%) 937 (33.56%) 49 (1.76%)	<0.001	–
**Preoperative treatment**, ***n*** **(%)**								
Beta blocker	0	3,887 (59.62%)	144 (52.55%)	1,032 (58.11%)	979 (59.66%)	1,718 (61.53%)	0.009	–
Statin	0	3,837 (58.85%)	157 (57.30%)	1,045 (58.84%)	1,024 (62.40%)	1,598 (57.23%)	0.008	–
Calcium channel blockers	0	1,298 (19.91%)	74 (27.01%)	417 (23.48%)	341 (20.78%)	462 (16.55%)	<0.001	–
Angiotensin-converting enzyme inhibitor	0	3,313 (50.81%)	108 (39.42%)	951 (53.55%)	853 (51.98%)	1,388 (49.71%)	<0.001	–

*BMI, body mass index*.

* *represent the result of non-parametric test*.

### Sensitivity Analyses

In order to test whether the results are robust, we did the following sensitivity analyses: (1) We verified the association between creatinine clearance and outcome in different subgroups; (2) The original database included the types of cardiac surgery. Therefore, we conducted a series of sensitivity analyses. First, we observe whether adjusting or not adjusting the type of cardiac surgery will have a significant impact on the results through different adjustment strategies; second, we observe whether the type of surgery will modify the association between creatinine clearance and the outcome through a stratified analysis; (3) Some patients (*n* = 57) received dialysis treatment. The creatinine clearance rate of these patients is inherently abnormal. Therefore, we excluded these 57 patients and performed the same analysis again to observe whether the 57 patients would affect the results. (4) Secondary diseases are also included in the original database. We, therefore, use different adjustment strategies to observe whether adjustment or non-adjustment of these secondary diseases will affect the results. In addition, we also use stratified analysis to observe whether secondary diseases will modify the association between creatinine clearance and outcome.

## Results

### Baseline Characteristics

After excluding patients with emergency cardiac surgery, we enrolled 6,520 patients for analyses, and 411 died during hospitalization. The average age of the patients was 63 years old, and ~68% of them were male. We compared the distribution of baseline data in different creatinine clearance groups (according to clinical stage), and the results are in Table 1. There was no statistically significant difference in myocardial infarction <90 days and chronic kidney disease requiring dialysis among the different groups. The highest mortality rate was observed in the Ccr <30 mL.min^−1^ group. The two groups with low Ccr were older. With the gradual decrease in Ccr, patients with low activity had worse heart function, a history of cardiac congestive failure, a history of thromboembolic events, and a greater occurrence of hypertension, peripheral vascular disease, valve disease, ischaemic stroke, and immunodeficiency.

### The Relationship Between Preoperative Ccr and Death During Hospitalization

We used a binary logistic regression model to evaluate the associations between preoperative Ccr and mortality (Table 2). In the non-adjusted model (no covariates were controlled), an increase of 1 ml/min of CC (calculated by CG) was associated with a 2% reduction in the risk of death. Similar results were also observed in the minimally adjusted model (adjusted only for age and sex) and fully adjusted model (we adjusted for age, sex, bmi, poor mobility, smoking status, left ventricular ejection fraction, hypertension, diabetes mellitus, dyslipidemia, myocardial infarction <90 days, history of coronary artery disease, history of cardiac congestive failure, history of thromboembolic event, peripheral vascular disease, valve disease, ischemic stroke, chronic pulmonary disease, immunodeficiency, chronic kidney disease requiring dialysis, new york heart association class 1,234, preoperative treatment beta blocker, statin, calcium channel blockers, angiotensin converting enzyme inhibitor and number of vessel-disease01234.) For the purpose of the sensitivity analysis, we additionally performed the following analysis. First, the Ccr calculated by the MDRD formula was analyzed with the same adjustment strategy, and the result was still the same as the CG result. Second, we converted Ccr from a continuous variable to a categorical variable based on clinical cut-off points and calculated the P for trend. The results show that the P for trend of the three models was still significant and was the same as the *P* value when Ccr is used as a continuous variable. However, different categories show non-equidistant changes which indicate the possibility of nonlinear relationships.

**Table 2:** The results of unvariate and multivariate analyses.

**Exposure**	**Non-adjusted model OR, 95%CI**	**Minimally-adjusted model OR, 95%CI**	**Fully-adjusted model OR, 95%CI**
Creatinine clearance (CG), mL.min^−1^	0.98 (0.98, 0.98) <0.0001	0.98 (0.97, 0.98) <0.0001	0.98 (0.98, 0.99) <0.0001
<30	Ref	Ref	Ref
≥30, <60	0.38 (0.27, 0.53) <0.0001	0.37 (0.27, 0.52) <0.0001	0.46 (0.33, 0.66) <0.0001
≥60, <80	0.20 (0.14, 0.29) <0.0001	0.21 (0.14, 0.30) <0.0001	0.30 (0.20, 0.45) <0.0001
≥80	0.13 (0.09, 0.19) <0.0001	0.15 (0.10, 0.22) <0.0001	0.25 (0.16, 0.39) <0.0001
*P* for trend	<0.0001	<0.0001	<0.0001
Creatinine clearance (MDRD), mL.min^−1^	0.98 (0.98, 0.98) <0.0001	0.98 (0.98, 0.99) <0.0001	0.99 (0.98, 0.99) <0.0001
<30	Ref	Ref	Ref
≥30, <60	0.39 (0.27, 0.57) <0.0001	0.37 (0.25, 0.53) <0.0001	0.44 (0.29, 0.65) <0.0001
≥60, <80	0.20 (0.14, 0.29) <0.0001	0.20 (0.14, 0.30) <0.0001	0.29 (0.19, 0.43) <0.0001
≥80	0.15 (0.10, 0.22) <0.0001	0.18 (0.12, 0.27) <0.0001	0.28 (0.19, 0.43) <0.0001
*P* for trend	<0.0001	<0.0001	<0.0001

*Non-adjusted model adjust for: None. Minimally-adjusted model: We only adjusted for age and sex*.

*Fully-adjusted model: We adjusted for all covariates presented Table 1*.

### Analyses of Nonlinear Relationships and Saturation or Threshold Effects

**Figure 2:**
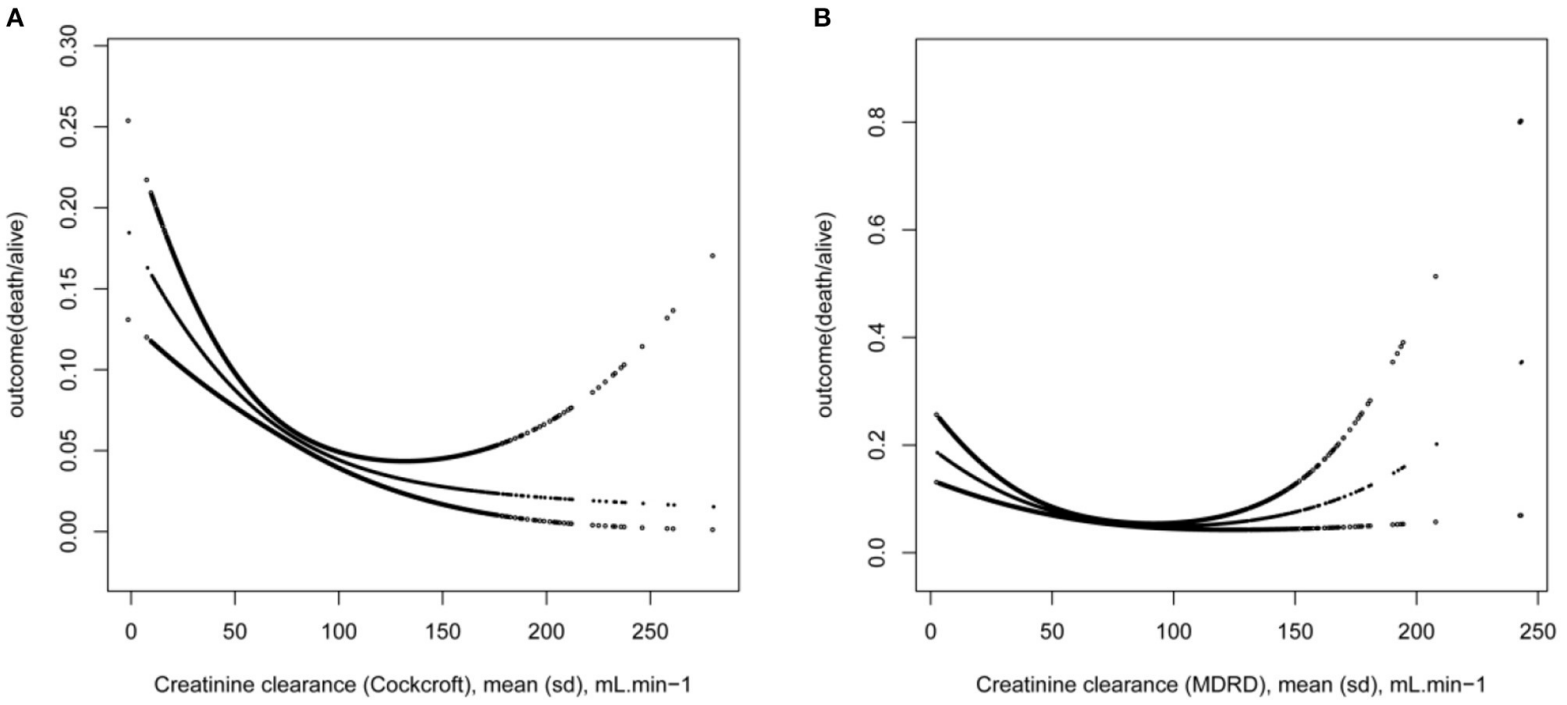
The non-linear on Ccr and outcome. **(A)** The outcome in CG formula, **(B)** The outcome in MDRD formula.

We found a nonlinear relationship between preoperative Ccr and death during hospitalization (Figure 2). By using a two-piecewise linear regression model, we calculated that the inflection point was 78.3 (in CG formula) and 73.5 (in MDRD formula). On the left side of the inflection point, the OR, 95%, and *P* values were 0.98, 0.98–0.99 and < 0.0001 (CG) and 0.99, 0.98–0.99 and < 0.0001 (MDRD), respectively. The decrease in creatinine clearance is accompanied by an increase in the risk of death. On the right side of the inflection point, the OR, 95%, and *P* value were 0.99, 0.99–1.00, and 0.1685 (CG) and 1.00, 0.99–1.01, and 0.5656 (MDRD). We could not determine a relationship between preoperative Ccr and death during hospitalization (Table 3).

**Table 3:** Nonlinearity further addressing using two-piecewise logistic models.

	**Creatinine clearance (CG), OR, 95%CI, *P* value**	**Creatinine clearance (MDRD), OR, 95%CI, *P* value**
Fitting model using standard binary logistic regression model	0.98 (0.98, 0.99) <0.0001	0.99 (0.98, 0.99) <0.0001
**Fitting model using two-piecewise logistic regression model**		
Inflection point	78.3	73.5
< Inflection point	0.98 (0.97, 0.99) <0.0001	0.98 (0.97, 0.99) <0.0001
> Inflection point	0.99 (0.99, 1.00) 0.1685	1.00 (0.99, 1.01) 0.5656
P for log likelyhood ratio	0.022	<0.001

*Covariates which were adjusted of the same with fully-adjusted model*.

**Figure 3:**
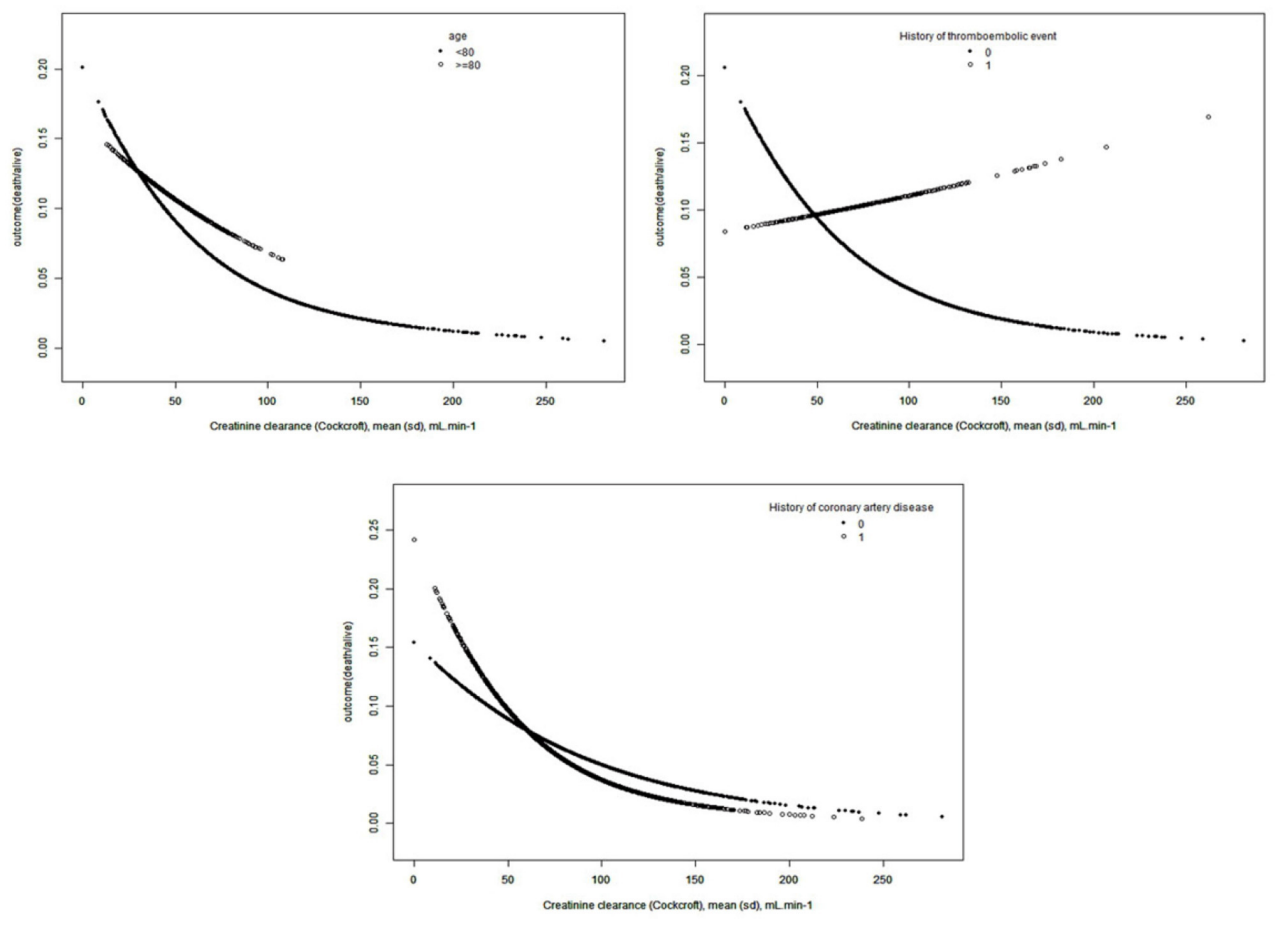
The non-linear on Ccr and outcome in stratified analysis.

In addition to this, we found that the associations between Cockcroft-Creatinine clearance and the outcome were different in person (<80 years old, with history of thromboembolic event and with history of coronary artery disease) (Figure 3). We calculated that the inflection point was 78.5 (<80 years), 30.8 (thromboembolic) and 56.6 (coronary artery disease).

On the left side of the inflection point, the OR, 95%, and *P* values were 0.98, 0.97–0.98 and < 0.0001 (<80 years old), 0.89, 0.83–0.97 and 0.0055 (thromboembolic) and 0.97, 0.95–0.98 and < 0.0001 (coronary artery disease), respectively. The decrease in creatinine clearance is accompanied by an increase in the risk of death. On the right side of the inflection point, the OR, 95%, and *P* value were 0.99, 0.98–1.00, and 0.0622 (<80 years old), 1.01, 1.00–1.03, and 0.0856 (thromboembolic) and 0.99, 0.98–1.00, and 0.1903 (coronary artery disease). We could not determine a relationship between preoperative Ccr and death during hospitalization (Table 4).

**Table 4:** Nonlinearity further addressing using two-piecewise logistic models in stratified analysis.

	**Creatinine clearance (CG), OR, 95%CI**, ***P*** **value**					
**Stratified analysis**	**Age <80**	**Age ≥80**	**History of thromboembolic event = 0**	**History of thromboembolic event = 1**	**History of coronary artery disease = 0**	**History of coronary artery disease = 1**
Fitting model using standard binary logistic regression model	0.98 (0.98, 0.99) <0.0001	0.99 (0.97, 1.00) 0.1127	0.98 (0.98, 0.99) <0.0001	1.00 (0.99, 1.01) 0.9874	0.99 (0.98, 0.99) <0.0001	0.98 (0.97, 0.99) <0.0001
**Fitting model using two-piecewise logistic regression model**						
Inflection point	78.5	31.9	78.9	30.8	83.8	56.6
< Inflection point	0.98 (0.97, 0.98) <0.0001	0.95 (0.87, 1.03) 0.1818	0.98 (0.97, 0.99) <0.0001	0.89 (0.83, 0.97) 0.0055	0.98 (0.97, 0.99) <0.0001	0.97 (0.95, 0.98) <0.0001
> Inflection point	0.99 (0.98, 1.00) 0.0622	0.99 (0.97, 1.01) 0.3782	0.99 (0.98, 1.00) 0.0610	1.01 (1.00, 1.03) 0.0856	0.99 (0.98, 1.01) 0.3310	0.99 (0.98, 1.00) 0.1903
P for log likelyhood ratio	0.031	0.331	0.078	0.005	0.085	0.035

*Covariates which were adjusted of the same with fully-adjusted model*.

### The Results of the Sensitivity Analyses

The results of subgroup analysis showed that the associations between Cockcroft-Creatinine clearance and MDRD-Creatinine clearance and the outcome were robust among all subgroups (except a history of a thromboembolic event, Chronic kidney disease requiring dialysis). The directions and values of odds ratio among different subgroups changed not significantly and with very small magnitude (<5%), and the 95% confidence interval are also stable (Supplementary Table 1).

We also found that he direction and size of the odds ratio of patients with a history of thrombosis have changed. However, for its relatively very small sample size, the result cannot be ruled out as a chancing finding. In addition, for patients with chronic kidney disease who require dialysis, the effect sizes of creatinine clearance are robust. Although these associations were not statistically significant, this is because the sample size is too small (only 57 patients).

We evaluated whether the type of cardiac surgery would affect the association between creatinine clearance and outcome through different adjustment strategies (Supplementary Table 2). Regardless of the type of cardiac surgery is adjusted, the direction of the effect sizes of creatinine clearance is the same, and the size and the 95% confidence interval range are almost unchanged. We also evaluated whether the type of cardiac surgery would affect the findings of two-piecewise linear models (Supplementary Tables 3, 4, Supplementary Figure 1). The results show that regardless of whether the type of cardiac surgery is adjusted for, the findings of two-pieceswise linear models and nonlinear association between creatinine clearance and outcome were virtually unchanged. Subgroup analysis showed that the effect size and confidence interval of creatinine clearance were robust among different cardiac operations (Supplementary Table 5).

To test whether the patients with dialysis can bias our findings, we excluded these 57 patients from this study, and the results did not change significantly (Supplementary Tables 6–8).

We followed the same strategy as for the type of surgery to investigate whether the secondary disease would affect the results. The results showed that there was no significant change in the results regardless of whether the secondary disease was adjusted or not. Even with the subgroup analysis of these secondary diseases, the results are all robust (Supplementary Tables 9–12).

## Discussion

Our secondary analysis of 6,520 patients that underwent elective cardiac surgery indicated a stable association between a gradual decrease in preoperative Ccr and an increased risk of death after adjustment for potential confounders. Additionally, a nonlinear relationship between preoperative Ccr and mortality during hospitalization was found. The linear decrease between Ccr and mortality reached a valley at a Ccr of 78.3 mL.min^−^1 (CG)/73.5 mL.min^−^1 (MDRD) (saturation effect). On the left side of the inflection point, an elevation in Ccr below 78.3 mL.min^−^1 (CG)/73.5 mL.min^−^1 (MDRD) decreased the risk of death occurrence. On the right side of the inflection point, we could not find a relationship between preoperative Ccr and mortality in hospitalization. However, This result is only applicable to people younger than 80 years old (CG). Besides, The linear decrease between Ccr and mortality reached a valley at a Ccr of 30.8 mL.min^−1^ (CG) in people with history of thromboembolic event /56.6 mL.min^−1^ (CG) in people with history of coronary artery disease. On the left side of the inflection point, an elevation in Ccr decreased the risk of death occurrence. On the right side of the inflection point, we could not find a relationship between preoperative Ccr and mortality in hospitalization.

Upon reviewing previous studies, we found that 5 papers had the same study outcome as ours. By studying 252 patients undergoing cardiac surgery under cardiopulmonary bypass, Jean-Michel Hougardy et al. found that baseline renal function can predict postoperative acute kidney injury and poor prognosis (15). Pablo Jorge Monjas and others found that renal function can be used as an important predictor of postoperative acute kidney injury. They studied 909 patients who underwent cardiac surgery with cardiopulmonary bypass (16). In the study of Takashi Yamauchi et al., a total of 1,484 consecutive nondialysis-dependent patients who underwent valvular operations using cardiopulmonary bypass revealed that renal function is an independent risk factor for acute kidney injury after surgery (17). Satoko Noguchi et al. reviewed 145 patients undergoing cardiac surgery with cardiopulmonary bypass and found that preoperative renal function could be a predictor of postoperative outcomes (18). Research done by Andrea L Axtell et al. shows that worse renal function before surgery and the duration of cardiopulmonary bypass are related to postoperative acute renal failure (19). Their findings are consistent with ours. However, none of these studies clarified the nonlinear association or indicated a safe range of Ccr before cardiac surgery. In general cardiac surgery patients, we not only confirmed that the worse the renal function before cardiac surgery is, the higher the postoperative mortality in hospitalization, but we also showed through non-linear correlation that there is no correlation between renal function and the risk of postoperative death with a Ccr above 78 mL.min^−1^ (CG) and 73.5 mL.min^−1^ (MDRD). In stratified analysis, we found that it need to establish a new evaluation model for cardiac surgery patients older than 80 years. People with history of thromboembolic event and with history of coronary artery disease, there is elevation in Ccr decreased the risk of postoperative in Ccr below 30.8 mL.min^−1^ (CG) and 56.6 mL.min^−1^ (CG).

Our research has some strengths. First, our research had a larger sample size than previous studies. Second, we observed the trends of the effect size of Ccr as continuous variables and categorical variables and tested the p for trend when Ccr was used as a categorical variable. The results were robust. Third, previous studies have shown that different Ccr calculation formulae have a greater impact on the results. Therefore, this study also analyzed the Ccr calculated by the CG and MDRD formulae and obtained consistent results. Fourth, we used the GAM to observe the nonlinear relationship. This method has obvious advantages because this model can handle nonparametric smoothing and will fit a regression spline to the data. It will help us to better discover the real relationship between exposure and outcome (20). Fifth, since this is an observational study and susceptible to potential confounding, and to minimize residual confounding, we used strict statistical adjustment. Sixth, a series of sensitivity analyses ensured the robustness of the results and minimized the possibility that our results are chancing findings.

Our study has some limitations. First, this study population consists only of French individuals, so its generalizability is limited. Second, we excluded patients with nonelective surgery; therefore, our results are not applicable for patients with this type of surgery. Third, this research is a secondary analysis; therefore, only the variables contained in the original database can be adjusted. Fourth, we only controlled for measurable confounders, not unmeasurable confounders.

## Data Availability Statement

Publicly available datasets were analyzed in this study. This data can be found here: Allyn J, et al. A Comparison of a Machine Learning Model with EuroSCORE II in predicting mortality after elective cardiac surgery: a decision curve analysis. *PLoS ONE*. (2017) 12(1):e0169772.

## Ethics Statement

The studies involving human participants were reviewed and approved by as an retrospective cohort study, the source research was approved by the Paris 7 University Ethics Committee and therefore waived informed consent. Written informed consent for participation was not required for this study in accordance with the national legislation and the institutional requirements.

## Author Contributions

YH contributed to conception and design of the study. KS organized the database. LL performed the statistical analysis. LC wrote the first draft of the manuscript. LC, KS, and LL wrote sections of the manuscript. All authors contributed to manuscript revision, read, and approved the submitted version.

## Funding

Ginkgo flavonoid aglycone affects the ox-LDL/LOX-1 system in the formation of atherosclerosis through NO-mediated oxidative stress, 201815815, Administration of Traditional Chinese Medicine in Guizhou Province. Project is not funded.

## Conflict of Interest

The authors declare that the research was conducted in the absence of any commercial or financial relationships that could be construed as a potential conflict of interest.

## Publisher's Note

All claims expressed in this article are solely those of the authors and do not necessarily represent those of their affiliated organizations, or those of the publisher, the editors and the reviewers. Any product that may be evaluated in this article, or claim that may be made by its manufacturer, is not guaranteed or endorsed by the publisher.
