# Supplementary material for: Preoperative Creatinine Clearance and Mortality of Elective Cardiac Surgery in Hospitalization: A Secondary Analysis
Source: Front Cardiovasc Med. 2022 Jan 27;8:712229. doi: 10.3389/fcvm.2021.712229 (PMC8830902; doi:10.3389/fcvm.2021.712229)

**Supplemental table 1: The results of subgroup analyses**

| Stratified variables | N | Creatinine clearance (Cockcroft), mean (sd), mL.min-1 | Creatinine clearance (MDRD), mean (sd), mL.min-1 |
| --- | --- | --- | --- |
| Age |  |  |  |
| ≤70 years old | 2302 | 0.98 (0.97, 0.99) <0.0001 | 0.98 (0.98, 0.99) <0.0001 |
| > 70 years old | 4218 | 0.98 (0.98, 0.99) <0.0001 | 0.98 (0.97, 0.99) <0.0001 |
| smoking status |  |  |  |
| non-smoker | 3159 | 0.98 (0.97, 0.98) <0.0001 | 0.98 (0.97, 0.98) <0.0001 |
| current smoker | 2327 | 0.98 (0.97, 0.99) <0.0001 | 0.98 (0.97, 0.99) <0.0001 |
| former smoker | 1009 | 0.98 (0.97, 0.99) 0.0004 | 0.98 (0.97, 0.99) 0.0004 |
| Ischemic stroke |  |  |  |
| No | 6056 | 0.98 (0.97, 0.98) <0.0001 | 0.98 (0.97, 0.98) <0.0001 |
| Yes | 464 | 0.99 (0.98, 1.00) 0.1642 | 0.99 (0.98, 1.01) 0.2948 |
| Poor mobility |  |  |  |
| No | 6341 | 0.98 (0.98, 0.98) <0.0001 | 0.98 (0.98, 0.98) <0.0001 |
| Yes | 179 | 0.98 (0.96, 1.00) 0.0155 | 0.98 (0.97, 1.00) 0.0454 |
| Peripheral vascular disease |  |  |  |
| No | 5615 | 0.98 (0.98, 0.98) <0.0001 | 0.98 (0.97, 0.98) <0.0001 |
| Yes | 905 | 0.98 (0.97, 0.99) <0.0001 | 0.98 (0.97, 0.99) 0.0001 |
| History of cardiac congestive failure |  |  |  |
| No | 5473 | 0.98 (0.97, 0.98) <0.0001 | 0.98 (0.97, 0.98) <0.0001 |
| Yes | 1047 | 0.99 (0.98, 0.99) 0.0003 | 0.99 (0.98, 1.00) 0.0026 |
| History of endocarditis |  |  |  |
| No | 6328 | 0.98 (0.98, 0.98) <0.0001 | 0.98 (0.97, 0.98) <0.0001 |
| Yes | 192 | 0.99 (0.97, 1.00) 0.0328 | 0.99 (0.97, 1.00) 0.0741 |
| History of thromboembolic event |  |  |  |
| No | 6167 | 0.98 (0.97, 0.98) <0.0001 | 0.98 (0.97, 0.98) <0.0001 |
| Yes | 353 | 1.00 (0.99, 1.01) 0.8104 | 1.00 (0.98, 1.01) 0.5508 |
| Chronic kidney disease requiring dialysis |  |  |  |
| No | 6463 | 0.98 (0.98, 0.98) <0.0001 | 0.98 (0.98, 0.98) <0.0001 |
| Yes | 57 | 0.98 (0.95, 1.02) 0.3051 | 0.99 (0.96, 1.02) 0.5350 |
| hypertension |  |  |  |
| No | 2842 | 0.97 (0.97, 0.98) <0.0001 | 0.97 (0.97, 0.98) <0.0001 |
| Yes | 3678 | 0.98 (0.98, 0.99) <0.0001 | 0.98 (0.98, 0.99) <0.0001 |
| History of coronary artery disease |  |  |  |
| No | 4024 | 0.98 (0.98, 0.99) <0.0001 | 0.98 (0.98, 0.99) <0.0001 |
| Yes | 2496 | 0.97 (0.97, 0.98) <0.0001 | 0.97 (0.97, 0.98) <0.0001 |
| Myocardial infarction < 90 days |  |  |  |
| No | 6080 | 0.98 (0.98, 0.98) <0.0001 | 0.98 (0.98, 0.98) <0.0001 |
| Yes | 440 | 0.98 (0.96, 0.99) 0.0020 | 0.98 (0.96, 0.99) 0.0069 |
| Previous cardiac surgery |  |  |  |
| No | 5893 | 0.98 (0.97, 0.98) <0.0001 | 0.98 (0.98, 0.99) <0.0001 |
| Yes | 627 | 0.98 (0.98, 0.99) <0.0001 | 0.98 (0.97, 0.99) <0.0001 |
| Dyslipidemia |  |  |  |
| No | 3232 | 0.98 (0.97, 0.98) <0.0001 | 0.98 (0.97, 0.98) <0.0001 |
| Yes | 3288 | 0.98 (0.97, 0.98) <0.0001 | 0.98 (0.97, 0.99) <0.0001 |
| Chronic obstructive pulmonary disease |  |  |  |
| No | 5878 | 0.98 (0.97, 0.98) <0.0001 | 0.98 (0.97, 0.98) <0.0001 |
| Yes | 642 | 0.99 (0.98, 1.00) 0.0178 | 0.99 (0.98, 1.00) 0.0581 |
| BMI, mean (sd), kg.m-2 group (tertile) |  |  |  |
| T1 (111.98 - 24.3) | 2167 | 0.98 (0.97, 0.98) <0.0001 | 0.98 (0.97, 0.99) <0.0001 |
| T2 (24.31 - 27.85) | 2160 | 0.98 (0.97, 0.99) <0.0001 | 0.98 (0.97, 0.99) <0.0001 |
| T3 (27.89 - 50.22) | 2174 | 0.98 (0.98, 0.99) <0.0001 | 0.98 (0.97, 0.99) <0.0001 |
| New York Heart Association class |  |  |  |
| 1 | 1374 | 0.98 (0.98, 0.99) 0.0003 | 0.98 (0.97, 0.99) 0.0005 |
| 2 | 603 | 0.96 (0.94, 0.98) 0.0004 | 0.97 (0.94, 0.99) 0.0038 |
| 3 | 2614 | 0.98 (0.97, 0.99) <0.0001 | 0.98 (0.97, 0.99) <0.0001 |
| 4 | 1929 | 0.98 (0.98, 0.99) <0.0001 | 0.98 (0.98, 0.99) <0.0001 |
| Congestive heart failure |  |  |  |
| No | 6265 | 0.98 (0.98, 0.98) <0.0001 | 0.98 (0.98, 0.99) <0.0001 |
| Yes | 255 | 0.98 (0.97, 0.99) 0.0002 | 0.98 (0.97, 0.99) 0.0027 |
| Antiplatelet therapy |  |  |  |
| None | 2716 | 0.98 (0.98, 0.99) <0.0001 | 0.98 (0.98, 0.99) <0.0001 |
| Aspirin only | 2666 | 0.98 (0.97, 0.98) <0.0001 | 0.98 (0.97, 0.98) <0.0001 |
| Others | 1138 | 0.98 (0.97, 0.99) <0.0001 | 0.98 (0.97, 0.99) <0.0001 |
| Beta blocker |  |  |  |
| Taking | 2633 | 0.98 (0.98, 0.99) <0.0001 | 0.98 (0.97, 0.99) <0.0001 |
| Not taking | 3887 | 0.98 (0.97, 0.98) <0.0001 | 0.98 (0.97, 0.98) <0.0001 |
| Anti-arrhythmic |  |  |  |
| Taking | 5640 | 0.98 (0.98, 0.98) <0.0001 | 0.98 (0.98, 0.99) <0.0001 |
| Not taking | 880 | 0.98 (0.97, 0.99) <0.0001 | 0.98 (0.96, 0.99) <0.0001 |
| Statin |  |  |  |
| Taking | 2683 | 0.98 (0.97, 0.98) <0.0001 | 0.98 (0.97, 0.98) <0.0001 |
| Not taking | 3837 | 0.98 (0.97, 0.99) <0.0001 | 0.98 (0.97, 0.99) <0.0001 |
| Angiotensin-converting enzyme inhibitor |  |  |  |
| Taking | 3207 | 0.98 (0.97, 0.98) <0.0001 | 0.98 (0.97, 0.98) <0.0001 |
| Not taking | 3313 | 0.98 (0.98, 0.99) <0.0001 | 0.98 (0.98, 0.99) <0.0001 |
| Calcium Channel Blockers |  |  |  |
| Taking | 5222 | 0.98 (0.97, 0.98) <0.0001 | 0.98 (0.97, 0.98) <0.0001 |
| Not taking | 1298 | 0.98 (0.98, 0.99) 0.0001 | 0.98 (0.97, 0.99) 0.0009 |

**Supplemental table 2：Results after unadjusted/adjusted type of heart surgery**

| Exposure | Adjust I | Adjust II |
| --- | --- | --- |
| Creatinine clearance (Cockcroft) | 0.99 (0.98, 0.99) <0.0001 | 0.99 (0.98, 0.99) <0.0001 |
| Q1 | 1.0 | 1.0 |
| Q2 | 0.57 (0.43, 0.75) <0.0001 | 0.56 (0.43, 0.74) <0.0001 |
| Q3 | 0.44 (0.31, 0.62) <0.0001 | 0.44 (0.31, 0.62) <0.0001 |
| Q4 | 0.44 (0.28, 0.67) 0.0002 | 0.44 (0.28, 0.68) 0.0002 |
| P for trend | <0.0001 | <0.0001 |
| Creatinine clearance (MDRD) | 0.99 (0.98, 0.99) <0.0001 | 0.99 (0.98, 0.99) <0.0001 |
| Q1 | 1.0 | 1.0 |
| Q2 | 0.58 (0.44, 0.76) 0.0001 | 0.58 (0.44, 0.77) 0.0001 |
| Q3 | 0.53 (0.39, 0.73) <0.0001 | 0.54 (0.39, 0.73) <0.0001 |
| Q4 | 0.60 (0.43, 0.84) 0.0027 | 0.61 (0.43, 0.85) 0.0039 |
| P for trend | 0.0002 | 0.0004 |

Adjust I：we adjusted for all covariates presented table 1

Adjust II：Adjust I + cardiac surgery （coronary artery surgery, valve surgery, aortic valve surgery, mitral valve surgery, tricuspid valve surgery, and thoracic aortic surgery）

**Supplemental table 3: The findings of two-piecewise linear model using different adjustment strategies.**

| Exposure: | Creatinine clearance (Cockcroft) | |
| --- | --- | --- |
|  | Adjust I | Adjust II |
| Fitting model by stardard logistic regression model | 0.99 (0.98, 0.99) <0.0001 | 0.99 (0.98, 0.99) <0.0001 |
| Fitting model by two-piecewise linear model |  |  |
| Inflection point | 77.8 | 76.2 |
| <  Inflection point | 0.98 (0.97, 0.99) <0.0001 | 0.98 (0.97, 0.99) <0.0001 |
| >  Inflection point | 1.00 (0.99, 1.00) 0.2982 | 1.00 (0.99, 1.00) 0.2784 |
| P for log likely ratio test | 0.012 | 0.010 |

Adjust I：we adjusted for all covariates presented table 1

Adjust II：Adjust I + cardiac surgery （coronary artery surgery, valve surgery, aortic valve surgery, mitral valve surgery, tricuspid valve surgery, and thoracic aortic surgery）

**Supplemental table 4: The findings of two-piecewise linear model using different adjustment strategies.**

| Exposure: | Creatinine clearance (MDRD) | |
| --- | --- | --- |
|  | Adjust I | Adjust II |
| Fitting model by stardard logistic regression model | 0.99 (0.98, 0.99) <0.0001 | 0.99 (0.98, 0.99) <0.0001 |
| Fitting model by two-piecewise linear model |  |  |
| Inflection point | 77.4 | 74.2 |
| <  Inflection point | 0.98 (0.97, 0.99) <0.0001 | 0.98 (0.97, 0.99) <0.0001 |
| >  Inflection point | 1.01 (1.00, 1.02) 0.1827 | 1.01(1.00, 1.01) 0.2224 |
| P for log likely ratio test | <0.001 | <0.001 |

Adjust I：we adjusted for all covariates presented table 1

Adjust II：Adjust I + cardiac surgery （coronary artery surgery, valve surgery, aortic valve surgery, mitral valve surgery, tricuspid valve surgery, and thoracic aortic surgery）

**Supplemental table 5: The subgroup analyses using type of cardiac operation as stratified variables**

| Stratified variables | N | Creatinine clearance (Cockcroft) | Creatinine clearance (MDRD) |
| --- | --- | --- | --- |
| Coronary surgery |  |  |  |
| No | 3393 | 0.98 (0.98, 0.99) <0.0001 | 0.98 (0.98, 0.99) <0.0001 |
| Yes | 3127 | 0.98 (0.97, 0.98) <0.0001 | 0.98 (0.97, 0.98) <0.0001 |
| Valve surgery |  |  |  |
| No | 2871 | 0.98 (0.97, 0.99) <0.0001 | 0.98 (0.97, 0.99) <0.0001 |
| Yes | 3649 | 0.98 (0.98, 0.98) <0.0001 | 0.98 (0.98, 0.99) <0.0001 |
| Aortic valve surgery |  |  |  |
| No | 4138 | 0.98 (0.97, 0.98) <0.0001 | 0.98 (0.97, 0.98) <0.0001 |
| Yes | 2382 | 0.98 (0.98, 0.99) <0.0001 | 0.98 (0.98, 0.99) <0.0001 |
| Mitral valve surgery |  |  |  |
| No | 5003 | 0.98 (0.98, 0.98) <0.0001 | 0.98 (0.97, 0.98) <0.0001 |
| Yes | 1517 | 0.98 (0.97, 0.99) <0.0001 | 0.98 (0.97, 0.99) <0.0001 |
| Tricuspid valve surgery |  |  |  |
| No | 5755 | 0.98 (0.98, 0.98) <0.0001 | 0.98 (0.98, 0.98) <0.0001 |
| Yes | 765 | 0.98 (0.97, 0.99) <0.0001 | 0.98 (0.97, 0.99) <0.0001 |
| Thoracic aorta surgery |  |  |  |
| No | 5951 | 0.98 (0.97, 0.98) <0.0001 | 0.98 (0.97, 0.98) <0.0001 |
| Yes | 569 | 0.98 (0.97, 0.99) <0.0001 | 0.98 (0.97, 0.99) 0.0016 |

**Supplemental 6: The results of sensitivity analysis obtained from all patientns or patients without dialysis**

| Exposure | All patients | Patients without dialysis |
| --- | --- | --- |
| Creatinine clearance (Cockcroft) | 0.99 (0.98, 0.99) <0.0001 | 0.98 (0.98, 0.99) <0.0001 |
| Q1 | 1.0 | 1.0 |
| Q2 | 0.57 (0.43, 0.75) <0.0001 | 0.55 (0.42, 0.73) <0.0001 |
| Q3 | 0.44 (0.31, 0.62) <0.0001 | 0.43 (0.30, 0.61) <0.0001 |
| Q4 | 0.44 (0.28, 0.67) 0.0002 | 0.42 (0.27, 0.65) <0.0001 |
| P for trend | <0.0001 | <0.0001 |
| Creatinine clearance (MDRD) | 0.99 (0.98, 0.99) <0.0001 | 0.99 (0.98, 0.99) <0.0001 |
| Q1 | 1.0 | 1.0 |
| Q2 | 0.58 (0.44, 0.76) 0.0001 | 0.57 (0.43, 0.76) 0.0001 |
| Q3 | 0.53 (0.39, 0.73) <0.0001 | 0.52 (0.38, 0.71) <0.0001 |
| Q4 | 0.60 (0.43, 0.84) 0.0027 | 0.59 (0.42, 0.82) 0.0021 |
| P for trend | 0.81 (0.73, 0.91) 0.0002 | 0.81 (0.72, 0.90) 0.0002 |

we adjusted for all covariates presented table 1

**Supplemental table 7: The findings of two-piecewise linear model obtained from all patientns or patients without dialysis**

| Exposure: | Creatinine clearance (Cockcroft) | |
| --- | --- | --- |
|  | All patients | Patients without dialysis |
| Fitting model by stardard logistic regression model | 0.99 (0.98, 0.99) <0.0001 | 0.98 (0.98, 0.99) <0.0001 |
| Fitting model by two-piecewise linear model |  |  |
| Inflection point | 77.8 | 77.8 |
| <  Inflection point | 0.98 (0.97, 0.99) <0.0001 | 0.98 (0.97, 0.99) <0.0001 |
| >  Inflection point | 1.00 (0.99, 1.00) 0.2982 | 1.00 (0.99, 1.00) 0.2675 |
| P for log likely ratio test | 0.012 | 0.011 |

We adjusted for all covariates presented table 1

**Supplemental table 8: The findings of two-piecewise linear model obtained from all patientns or patients without dialysis**

| Exposure: | Creatinine clearance (MDRD) | |
| --- | --- | --- |
|  | Adjust I | Adjust II |
| Fitting model by stardard logistic regression model | 0.99 (0.98, 0.99) <0.0001 | 0.99 (0.98, 0.99) <0.0001 |
| Fitting model by two-piecewise linear model |  |  |
| Inflection point | 77.4 | 77.3 |
| <  Inflection point | 0.98 (0.97, 0.99) <0.0001 | 0.98 (0.97, 0.99) <0.0001 |
| >  Inflection point | 1.01 (1.00, 1.02) 0.1827 | 1.01(1.00, 1.02) 0.1605 |
| P for log likely ratio test | <0.001 | <0.001 |

We adjusted for all covariates presented table 1

**Supplemental table 9：Results after unadjusted/adjusted secondary diseases**

| Exposure | Adjust I | Adjust II |
| --- | --- | --- |
| Creatinine clearance (Cockcroft) | 0.99 (0.98, 0.99) <0.0001 | 0.99 (0.98, 0.99) <0.0001 |
| Q1 | 1.0 | 1.0 |
| Q2 | 0.57 (0.43, 0.75) <0.0001 | 0.61 (0.46, 0.80) 0.0004 |
| Q3 | 0.44 (0.31, 0.62) <0.0001 | 0.48 (0.34, 0.68) <0.0001 |
| Q4 | 0.44 (0.28, 0.67) 0.0002 | 0.50 (0.32, 0.77) 0.0016 |
| P for trend | <0.0001 | <0.0001 |
| Creatinine clearance (MDRD) | 0.99 (0.98, 0.99) <0.0001 | 0.99 (0.98, 0.99) <0.0001 |
| Q1 | 1.0 | 1.0 |
| Q2 | 0.58 (0.44, 0.76) 0.0001 | 0.63 (0.47, 0.83) 0.0012 |
| Q3 | 0.53 (0.39, 0.73) <0.0001 | 0.58 (0.42, 0.80) 0.0007 |
| Q4 | 0.60 (0.43, 0.84) 0.0027 | 0.67 (0.48, 0.94) 0.0193 |
| P for trend | 0.0002 | 0.0034 |

Adjust I：we adjusted for all covariates presented table 1

Adjust II：Adjust I + secondary diseases (atrial fibrillation, congestive heart failure, active endocarditis, critical preoperative state)

**Supplemental table 10: The findings of two-piecewise linear model using different adjustment strategies.**

| Exposure: | Creatinine clearance (Cockcroft) | |
| --- | --- | --- |
|  | Adjust I | Adjust II |
| Fitting model by stardard logistic regression model | 0.99 (0.98, 0.99) <0.0001 | 0.99 (0.98, 0.99) <0.0001 |
| Fitting model by two-piecewise linear model |  |  |
| Inflection point | 77.8 | 76.3 |
| <  Inflection point | 0.98 (0.97, 0.99) <0.0001 | 0.98 (0.97, 0.99) <0.0001 |
| >  Inflection point | 1.00 (0.99, 1.00) 0.2982 | 1.00 (0.99, 1.00) 0.3926 |
| P for log likely ratio test | 0.012 | 0.022 |

Adjust I：we adjusted for all covariates presented table 1

Adjust II：Adjust I + secondary diseases (atrial fibrillation, congestive heart failure, active endocarditis, critical preoperative state)

**Supplemental table 11: The findings of two-piecewise linear model using different adjustment strategies.**

| Exposure: | Creatinine clearance (MDRD) | |
| --- | --- | --- |
|  | Adjust I | Adjust II |
| Fitting model by stardard logistic regression model | 0.99 (0.98, 0.99) <0.0001 | 0.99 (0.98, 0.99) <0.0001 |
| Fitting model by two-piecewise linear model |  |  |
| Inflection point | 77.4 | 77.5 |
| <  Inflection point | 0.98 (0.97, 0.99) <0.0001 | 0.98 (0.97, 0.99) <0.0001 |
| >  Inflection point | 1.01 (1.00, 1.02) 0.1827 | 1.01(1.00, 1.02) 0.1360 |
| P for log likely ratio test | <0.001 | <0.001 |

Adjust I：we adjusted for all covariates presented table 1

Adjust II：Adjust I + secondary diseases (atrial fibrillation, congestive heart failure, active endocarditis, critical preoperative state)

**Supplemental table 12: The subgroup analyses using secondary diseases as stratified variables**

| Stratified variables | N | Creatinine clearance (Cockcroft) | Creatinine clearance (MDRD) |
| --- | --- | --- | --- |
| Congestive heart failure |  |  |  |
| 0 | 6265 | 0.98 (0.98, 0.98) <0.0001 | 0.98 (0.98, 0.99) <0.0001 |
| 1 | 255 | 0.98 (0.97, 0.99) 0.0002 | 0.98 (0.97, 0.99) 0.0027 |
| Active endocarditis |  |  |  |
| 0 | 6318 | 0.98 (0.98, 0.98) <0.0001 | 0.98 (0.98, 0.98) <0.0001 |
| 1 | 202 | 0.99 (0.97, 1.00) 0.0110 | 0.98 (0.97, 1.00) 0.0133 |
| Critical preoperative state |  |  |  |
| 0 | 6413 | 0.98 (0.98, 0.98) <0.0001 | 0.98 (0.98, 0.99) <0.0001 |
| 1 | 107 | 0.98 (0.97, 1.00) 0.0289 | 0.98 (0.96, 1.00) 0.0272 |
| Atrial fibrillation |  |  |  |
| Sinus rhythm | 5744 | 0.98 (0.98, 0.98) <0.0001 | 0.98 (0.97, 0.98) <0.0001 |
| Yes | 776 | 0.98 (0.96, 1.00) <0.0001 | 0.98 (0.97, 1.00) <0.0001 |

**Supplemental table 13: The results of subgroup analyses using age as stratified variables**

|  | Creatinine clearance (CG), OR, 95%CI, P value | | | Creatinine clearance (MDRD), OR, 95%CI, P value | | |
| --- | --- | --- | --- | --- | --- | --- |
| Age | <80 | >=80 | Total | <80 | >=80 | Total |
| Fitting model using standard binary logistic regression model | 1.0 (1.0, 1.0) <0.001 | 1.0 (1.0, 1.0) 0.113 | 1.0 (1.0, 1.0) <0.001 | 1.0 (1.0, 1.0) <0.001 | 1.0 (1.0, 1.0) 0.594 | 1.0 (1.0, 1.0) <0.001 |
| Fitting model using two-piecewise logistic regression model |  |  |  |  |  |  |
| Inflection point | 78.5 | 31.9 | 78.7 | 77.6 | 47.5 | 79.2 |
| < Inflection point | 1.0 (1.0, 1.0) <0.001 | 0.9 (0.9, 1.0) 0.182 | 1.0 (1.0, 1.0) <0.001 | 1.0 (1.0, 1.0) <0.001 | 1.0 (0.9, 1.0) 0.223 | 1.0 (1.0, 1.0) <0.001 |
| > Inflection point | 1.0 (1.0, 1.0) 0.062 | 1.0 (1.0, 1.0) 0.378 | 1.0 (1.0, 1.0) 0.032 | 1.0 (1.0, 1.0) 0.870 | 1.0 (1.0, 1.0) 0.755 | 1.0 (1.0, 1.0) 0.993 |
| P for log likelyhood ratio | 0.031 | 0.331 | 0.051 | <0.001 | 0.283 | 0.002 |

**Supplemental table 14: The results of subgroup analyses using sex as stratified variables**

|  | Creatinine clearance (CG), OR, 95%CI, P value | | | Creatinine clearance (MDRD), OR, 95%CI, P value | | |
| --- | --- | --- | --- | --- | --- | --- |
| Sex | 1 | 2 | Total | 1 | 2 | Total |
| Fitting model using standard binary logistic regression model | 1.0 (1.0, 1.0) <0.001 | 1.0 (1.0, 1.0) <0.001 | 1.0 (1.0, 1.0) <0.001 | 1.0 (1.0, 1.0) <0.001 | 1.0 (1.0, 1.0) <0.001 | 1.0 (1.0, 1.0) <0.001 |
| Fitting model using two-piecewise logistic regression model |  |  |  |  |  |  |
| Inflection point | 79.2 | 124 | 78.3 | 83.9 | 70.5 | 73.5 |
| < Inflection point | 1.0 (1.0, 1.0) <0.001 | 1.0 (1.0, 1.0) <0.001 | 1.0 (1.0, 1.0) <0.001 | 1.0 (1.0, 1.0) <0.001 | 1.0 (1.0, 1.0) <0.001 | 1.0 (1.0, 1.0) <0.001 |
| > Inflection point | 1.0 (1.0, 1.0) 0.467 | 0.0 (0.0, Inf) 0.987 | 1.0 (1.0, 1.0) 0.168 | 1.0 (1.0, 1.0) 0.180 | 1.0 (1.0, 1.0) 0.933 | 1.0 (1.0, 1.0) 0.566 |
| P for log likelyhood ratio | 0.021 | 0.068 | 0.022 | 0.001 | 0.052 | <0.001 |

**Supplemental table 15: The results of subgroup analyses using smoking status as stratified variables**

|  | Creatinine clearance (CG), OR, 95%CI, P value | | | | Creatinine clearance (MDRD),  OR, 95%CI, P value | | | |
| --- | --- | --- | --- | --- | --- | --- | --- | --- |
| Smoking status | non-smoker | current smoker | former smoker | Total | non-smoker | current smoker | former smoker | Total |
| Fitting model using standard binary logistic regression model | 1.0 (1.0, 1.0) <0.001 | 1.0 (1.0, 1.0) <0.001 | 1.0 (1.0, 1.0) 0.002 | 1.0 (1.0, 1.0) <0.001 | 1.0 (1.0, 1.0) <0.001 | 1.0 (1.0, 1.0) 0.004 | 1.0 (1.0, 1.0) 0.021 | 1.0 (1.0, 1.0) <0.001 |
| Fitting model using two-piecewise logistic regression model |  |  |  |  |  |  |  |  |
| Inflection point | 75.7 | 38.9 | 51.2 | 78.3 | 73.1 | 108.7 | 44.7 | 73.5 |
| < Inflection point | 1.0 (1.0, 1.0) <0.001 | 1.0 (1.0, 1.1) 0.391 | 0.9 (0.9, 1.0) <0.001 | 1.0 (1.0, 1.0) <0.001 | 1.0 (1.0, 1.0) <0.001 | 1.0 (1.0, 1.0) <0.001 | 0.9 (0.9, 1.0) <0.001 | 1.0 (1.0, 1.0) <0.001 |
| > Inflection point | 1.0 (1.0, 1.0) 0.396 | 1.0 (1.0, 1.0) <0.001 | 1.0 (1.0, 1.0) 0.400 | 1.0 (1.0, 1.0) 0.168 | 1.0 (1.0, 1.0) 0.261 | 1.0 (1.0, 1.0) 0.173 | 1.0 (1.0, 1.0) 0.817 | 1.0 (1.0, 1.0) 0.566 |
| P for log likelyhood ratio | 0.060 | 0.066 | 0.008 | 0.022 | 0.001 | 0.061 | 0.005 | <0.001 |

**Supplemental table 16: The results of subgroup analyses using BMI as stratified variables**

|  | Creatinine clearance (CG), OR, 95%CI, P value | | | | | Creatinine clearance (MDRD),  OR, 95%CI, P value | | | | | |
| --- | --- | --- | --- | --- | --- | --- | --- | --- | --- | --- | --- |
| BMI, mean (sd), kg.m-2 | <24 | >=24, <30 | >=30, <35 | >=35 | Total | | <24 | >=24, <30 | >=30, <35 | >=35 | Total |
| Fitting model using standard binary logistic regression model | 1.0 (1.0, 1.0) <0.001 | 1.0 (1.0, 1.0) <0.001 | 1.0 (1.0, 1.0) 0.705 | 1.0 (0.9, 1.0) 0.003 | 1.0 (1.0, 1.0) <0.001 | | 1.0 (1.0, 1.0) 0.006 | 1.0 (1.0, 1.0) <0.001 | 1.0 (1.0, 1.0) 0.795 | 1.0 (0.9, 1.0) 0.004 | 1.0 (1.0, 1.0) <0.001 |
| Fitting model using two-piecewise logistic regression model |  |  |  |  |  | |  |  |  |  |  |
| Inflection point | 26 | 79 | 152.1 | 124 | 78.3 | | 83.1 | 73.3 | 59.3 | 92 | 72.9 |
| < Inflection point | 1.0 (1.0, 1.1) 0.293 | 1.0 (1.0, 1.0) <0.001 | 1.0 (1.0, 1.0) 0.890 | 1.0 (1.0, 1.0) 0.014 | 1.0 (1.0, 1.0) <0.001 | | 1.0 (1.0, 1.0) <0.001 | 1.0 (1.0, 1.0) <0.001 | 1.0 (1.0, 1.0) 0.586 | 1.0 (0.9, 1.0) 0.028 | 1.0 (1.0, 1.0) <0.001 |
| > Inflection point | 1.0 (1.0, 1.0) <0.001 | 1.0 (1.0, 1.0) 0.683 | 0.0 (0.0, Inf) 0.988 | 0.0 (0.0, Inf) 0.982 | 1.0 (1.0, 1.0) 0.169 | | 1.0 (1.0, 1.0) 0.285 | 1.0 (1.0, 1.0) 0.421 | 1.0 (1.0, 1.0) 0.854 | 0.0 (0.0, Inf) 0.994 | 1.0 (1.0, 1.0) 0.574 |
| P for log likelyhood ratio | 0.042 | 0.059 | 0.184 | 0.148 | 0.031 | | 0.012 | 0.003 | 0.638 | 0.205 | <0.001 |

**Supplemental table 17: The results of subgroup analyses using poor mobility as stratified variables**

|  | Creatinine clearance (CG), OR, 95%CI, P value | | | Creatinine clearance (MDRD),  OR, 95%CI, P value | | |
| --- | --- | --- | --- | --- | --- | --- |
| Poor mobility | 0 | 1 | Total | 0 | 1 | Total |
| Fitting model using standard binary logistic regression model | 1.0 (1.0, 1.0) <0.001 | 1.0 (1.0, 1.0) 0.780 | 1.0 (1.0, 1.0) <0.001 | 1.0 (1.0, 1.0) <0.001 | 1.0 (1.0, 1.0) 0.833 | 1.0 (1.0, 1.0) <0.001 |
| Fitting model using two-piecewise logistic regression model |  |  |  |  |  |  |
| Inflection point | 78.4 | 93.6 | 78.3 | 73.7 | 18.6 | 73.5 |
| < Inflection point | 1.0 (1.0, 1.0) <0.001 | 1.0 (1.0, 1.1) 0.447 | 1.0 (1.0, 1.0) <0.001 | 1.0 (1.0, 1.0) <0.001 | 0.7 (0.5, 1.1) 0.147 | 1.0 (1.0, 1.0) <0.001 |
| > Inflection point | 1.0 (1.0, 1.0) 0.195 | 0.0 (0.0, Inf) 0.995 | 1.0 (1.0, 1.0) 0.168 | 1.0 (1.0, 1.0) 0.484 | 1.0 (1.0, 1.1) 0.342 | 1.0 (1.0, 1.0) 0.566 |
| P for log likelyhood ratio | 0.013 | 0.034 | 0.022 | <0.001 | 0.150 | <0.001 |

**Supplemental table 18: The results of subgroup analyses using hypertension as stratified variables**

|  | Creatinine clearance (CG), OR, 95%CI, P value | | | Creatinine clearance (MDRD),  OR, 95%CI, P value | | |
| --- | --- | --- | --- | --- | --- | --- |
| Hypertension | 0 | 1 | Total | 0 | 1 | Total |
| Fitting model using standard binary logistic regression model | 1.0 (1.0, 1.0) <0.001 | 1.0 (1.0, 1.0) <0.001 | 1.0 (1.0, 1.0) <0.001 | 1.0 (1.0, 1.0) <0.001 | 1.0 (1.0, 1.0) 0.001 | 1.0 (1.0, 1.0) <0.001 |
| Fitting model using two-piecewise logistic regression model |  |  |  |  |  |  |
| Inflection point | 82.6 | 75.9 | 78.3 | 80.6 | 70.2 | 73.5 |
| < Inflection point | 1.0 (1.0, 1.0) <0.001 | 1.0 (1.0, 1.0) <0.001 | 1.0 (1.0, 1.0) <0.001 | 1.0 (1.0, 1.0) <0.001 | 1.0 (1.0, 1.0) <0.001 | 1.0 (1.0, 1.0) <0.001 |
| > Inflection point | 1.0 (1.0, 1.0) 0.159 | 1.0 (1.0, 1.0) 0.679 | 1.0 (1.0, 1.0) 0.168 | 1.0 (1.0, 1.0) 0.531 | 1.0 (1.0, 1.0) 0.464 | 1.0 (1.0, 1.0) 0.566 |
| P for log likelyhood ratio | 0.085 | 0.041 | 0.022 | 0.001 | 0.009 | <0.001 |

**Supplemental table 19: The results of subgroup analyses using diabetes mellitus as stratified variables**

|  | Creatinine clearance (CG), OR, 95%CI, P value | | | Creatinine clearance (MDRD),  OR, 95%CI, P value | | |
| --- | --- | --- | --- | --- | --- | --- |
| Diabetes mellitus | 0 | 1 | Total | 0 | 1 | Total |
| Fitting model using standard binary logistic regression model | 1.0 (1.0, 1.0) <0.001 | 1.0 (1.0, 1.0) 0.002 | 1.0 (1.0, 1.0) <0.001 | 1.0 (1.0, 1.0) <0.001 | 1.0 (1.0, 1.0) 0.042 | 1.0 (1.0, 1.0) <0.001 |
| Fitting model using two-piecewise logistic regression model |  |  |  |  |  |  |
| Inflection point | 77.7 | 29 | 78.3 | 74.7 | 70.2 | 73.5 |
| < Inflection point | 1.0 (1.0, 1.0) <0.001 | 1.0 (1.0, 1.1) 0.576 | 1.0 (1.0, 1.0) <0.001 | 1.0 (1.0, 1.0) <0.001 | 1.0 (1.0, 1.0) 0.029 | 1.0 (1.0, 1.0) <0.001 |
| > Inflection point | 1.0 (1.0, 1.0) 0.333 | 1.0 (1.0, 1.0) 0.002 | 1.0 (1.0, 1.0) 0.168 | 1.0 (1.0, 1.0) 0.395 | 1.0 (1.0, 1.0) 0.952 | 1.0 (1.0, 1.0) 0.566 |
| P for log likelyhood ratio | 0.008 | 0.246 | 0.022 | <0.001 | 0.242 | <0.001 |

**Supplemental table 20: The results of subgroup analyses using dyslipidemia as stratified variables**

|  | Creatinine clearance (CG), OR, 95%CI, P value | | | Creatinine clearance (MDRD),  OR, 95%CI, P value | | |
| --- | --- | --- | --- | --- | --- | --- |
| Dyslipidemia | 0 | 1 | Total | 0 | 1 | Total |
| Fitting model using standard binary logistic regression model | 1.0 (1.0, 1.0) <0.001 | 1.0 (1.0, 1.0) <0.001 | 1.0 (1.0, 1.0) <0.001 | 1.0 (1.0, 1.0) <0.001 | 1.0 (1.0, 1.0) 0.004 | 1.0 (1.0, 1.0) <0.001 |
| Fitting model using two-piecewise logistic regression model |  |  |  |  |  |  |
| Inflection point | 79.1 | 76.3 | 78.3 | 85.8 | 62 | 73.5 |
| < Inflection point | 1.0 (1.0, 1.0) <0.001 | 1.0 (1.0, 1.0) <0.001 | 1.0 (1.0, 1.0) <0.001 | 1.0 (1.0, 1.0) <0.001 | 1.0 (1.0, 1.0) <0.001 | 1.0 (1.0, 1.0) <0.001 |
| > Inflection point | 1.0 (1.0, 1.0) 0.317 | 1.0 (1.0, 1.0) 0.260 | 1.0 (1.0, 1.0) 0.168 | 1.0 (1.0, 1.0) 0.326 | 1.0 (1.0, 1.0) 0.630 | 1.0 (1.0, 1.0) 0.566 |
| P for log likelyhood ratio | 0.037 | 0.242 | 0.022 | 0.002 | 0.005 | <0.001 |

**Supplemental table 21: The results of subgroup analyses using myocardial infarction < 90 days as stratified variables**

|  | Creatinine clearance (CG), OR, 95%CI, P value | | | Creatinine clearance (MDRD),  OR, 95%CI, P value | | |
| --- | --- | --- | --- | --- | --- | --- |
| Myocardial infarction < 90 days | 0 | 1 | Total | 0 | 1 | Total |
| Fitting model using standard binary logistic regression model | 1.0 (1.0, 1.0) <0.001 | 1.0 (1.0, 1.0) 0.280 | 1.0 (1.0, 1.0) <0.001 | 1.0 (1.0, 1.0) <0.001 | 1.0 (1.0, 1.0) 0.693 | 1.0 (1.0, 1.0) <0.001 |
| Fitting model using two-piecewise logistic regression model |  |  |  |  |  |  |
| Inflection point | 79 | 135.5 | 78.3 | 73.5 | 93.7 | 73.5 |
| < Inflection point | 1.0 (1.0, 1.0) <0.001 | 1.0 (1.0, 1.0) 0.134 | 1.0 (1.0, 1.0) <0.001 | 1.0 (1.0, 1.0) <0.001 | 1.0 (1.0, 1.0) 0.261 | 1.0 (1.0, 1.0) <0.001 |
| > Inflection point | 1.0 (1.0, 1.0) 0.186 | 1.1 (1.0, 1.3) 0.128 | 1.0 (1.0, 1.0) 0.168 | 1.0 (1.0, 1.0) 0.594 | 1.0 (1.0, 1.1) 0.189 | 1.0 (1.0, 1.0) 0.566 |
| P for log likelyhood ratio | 0.029 | 0.157 | 0.022 | <0.001 | 0.166 | <0.001 |

**Supplemental table 22: The results of subgroup analyses using peripheral vascular disease as stratified variables**

|  | Creatinine clearance (CG), OR, 95%CI, P value | | | Creatinine clearance (MDRD),  OR, 95%CI, P value | | |
| --- | --- | --- | --- | --- | --- | --- |
| Peripheral vascular disease | 0 | 1 | Total | 0 | 1 | Total |
| Fitting model using standard binary logistic regression model | 1.0 (1.0, 1.0) <0.001 | 1.0 (1.0, 1.0) 0.005 | 1.0 (1.0, 1.0) <0.001 | 1.0 (1.0, 1.0) <0.001 | 1.0 (1.0, 1.0) 0.008 | 1.0 (1.0, 1.0) <0.001 |
| Fitting model using two-piecewise logistic regression model |  |  |  |  |  |  |
| Inflection point | 76.3 | 80.4 | 78.3 | 73.1 | 79.5 | 73.5 |
| < Inflection point | 1.0 (1.0, 1.0) <0.001 | 1.0 (1.0, 1.0) <0.001 | 1.0 (1.0, 1.0) <0.001 | 1.0 (1.0, 1.0) <0.001 | 1.0 (1.0, 1.0) <0.001 | 1.0 (1.0, 1.0) <0.001 |
| > Inflection point | 1.0 (1.0, 1.0) 0.065 | 1.0 (1.0, 1.0) 0.624 | 1.0 (1.0, 1.0) 0.168 | 1.0 (1.0, 1.0) 0.894 | 1.0 (1.0, 1.0) 0.177 | 1.0 (1.0, 1.0) 0.566 |
| P for log likelyhood ratio | 0.076 | 0.068 | 0.022 | 0.004 | 0.014 | <0.001 |

**Supplemental table 23: The results of subgroup analyses using congestive heart failure as stratified variables**

|  | Creatinine clearance (CG), OR, 95%CI, P value | | | Creatinine clearance (MDRD),  OR, 95%CI, P value | | |
| --- | --- | --- | --- | --- | --- | --- |
| Congestive heart failure | 0 | 1 | Total | 0 | 1 | Total |
| Fitting model using standard binary logistic regression model | 1.0 (1.0, 1.0) <0.001 | 1.0 (1.0, 1.0) 0.007 | 1.0 (1.0, 1.0) <0.001 | 1.0 (1.0, 1.0) <0.001 | 1.0 (1.0, 1.0) 0.011 | 1.0 (1.0, 1.0) <0.001 |
| Fitting model using two-piecewise logistic regression model |  |  |  |  |  |  |
| Inflection point | 72.6 | 54.1 | 78.2 | 74.5 | 32.8 | 73.6 |
| < Inflection point | 1.0 (1.0, 1.0) <0.001 | 1.0 (1.0, 1.0) 0.801 | 1.0 (1.0, 1.0) <0.001 | 1.0 (1.0, 1.0) <0.001 | 1.0 (0.9, 1.1) 0.747 | 1.0 (1.0, 1.0) <0.001 |
| > Inflection point | 1.0 (1.0, 1.0) 0.150 | 1.0 (0.9, 1.0) 0.005 | 1.0 (1.0, 1.0) 0.181 | 1.0 (1.0, 1.0) 0.455 | 1.0 (1.0, 1.0) 0.014 | 1.0 (1.0, 1.0) 0.633 |
| P for log likelyhood ratio | 0.020 | 0.046 | 0.024 | <0.001 | 0.382 | <0.001 |

**Supplemental table 24: The results of subgroup analyses using history of thromboembolic event as stratified variables**

|  | Creatinine clearance (CG), OR, 95%CI, P value | | | Creatinine clearance (MDRD),  OR, 95%CI, P value | | |
| --- | --- | --- | --- | --- | --- | --- |
| History of thromboembolic event | 0 | 1 | Total | 0 | 1 | Total |
| Fitting model using standard binary logistic regression model | 1.0 (1.0, 1.0) <0.001 | 1.0 (1.0, 1.0) 0.987 | 1.0 (1.0, 1.0) <0.001 | 1.0 (1.0, 1.0) <0.001 | 1.0 (1.0, 1.0) 0.878 | 1.0 (1.0, 1.0) <0.001 |
| Fitting model using two-piecewise logistic regression model |  |  |  |  |  |  |
| Inflection point | 78.9 | 30.8 | 78.3 | 79.2 | 113.6 | 73.5 |
| < Inflection point | 1.0 (1.0, 1.0) <0.001 | 0.9 (0.8, 1.0) 0.005 | 1.0 (1.0, 1.0) <0.001 | 1.0 (1.0, 1.0) <0.001 | 1.0 (1.0, 1.0) 0.555 | 1.0 (1.0, 1.0) <0.001 |
| > Inflection point | 1.0 (1.0, 1.0) 0.061 | 1.0 (1.0, 1.0) 0.086 | 1.0 (1.0, 1.0) 0.168 | 1.0 (1.0, 1.0) 0.299 | 0.9 (0.7, 1.1) 0.404 | 1.0 (1.0, 1.0) 0.566 |
| P for log likelyhood ratio | 0.078 | 0.005 | 0.022 | <0.001 | 0.120 | <0.001 |

**Supplemental table 25: The results of subgroup analyses using history of coronary artery disease as stratified variables**

|  | Creatinine clearance (CG), OR, 95%CI, P value | | | Creatinine clearance (MDRD),  OR, 95%CI, P value | | |
| --- | --- | --- | --- | --- | --- | --- |
| History of coronary artery disease | 0 | 1 | Total | 0 | 1 | Total |
| Fitting model using standard binary logistic regression model | 1.0 (1.0, 1.0) <0.001 | 1.0 (1.0, 1.0) <0.001 | 1.0 (1.0, 1.0) <0.001 | 1.0 (1.0, 1.0) <0.001 | 1.0 (1.0, 1.0) <0.001 | 1.0 (1.0, 1.0) <0.001 |
| Fitting model using two-piecewise logistic regression model |  |  |  |  |  |  |
| Inflection point | 83.8 | 56.6 | 78.3 | 73 | 42.4 | 73.5 |
| < Inflection point | 1.0 (1.0, 1.0) <0.001 | 1.0 (1.0, 1.0) <0.001 | 1.0 (1.0, 1.0) <0.001 | 1.0 (1.0, 1.0) <0.001 | 0.9 (0.9, 1.0) <0.001 | 1.0 (1.0, 1.0) <0.001 |
| > Inflection point | 1.0 (1.0, 1.0) 0.331 | 1.0 (1.0, 1.0) 0.190 | 1.0 (1.0, 1.0) 0.168 | 1.0 (1.0, 1.0) 0.665 | 1.0 (1.0, 1.0) 0.220 | 1.0 (1.0, 1.0) 0.566 |
| P for log likelyhood ratio | 0.085 | 0.035 | 0.022 | 0.005 | 0.012 | <0.001 |

**Supplemental table 26: The results of subgroup analyses using valve disease as stratified variables**

|  | Creatinine clearance (CG), OR, 95%CI, P value | | | Creatinine clearance (MDRD),  OR, 95%CI, P value | | |
| --- | --- | --- | --- | --- | --- | --- |
| Valve disease | 0 | 1 | Total | 0 | 1 | Total |
| Fitting model using standard binary logistic regression model | 1.0 (1.0, 1.0) 0.003 | 1.0 (1.0, 1.0) <0.001 | 1.0 (1.0, 1.0) <0.001 | 1.0 (1.0, 1.0) 0.034 | 1.0 (1.0, 1.0) <0.001 | 1.0 (1.0, 1.0) <0.001 |
| Fitting model using two-piecewise logistic regression model |  |  |  |  |  |  |
| Inflection point | 100.2 | 90.8 | 78.3 | 77.4 | 72.3 | 73.5 |
| < Inflection point | 1.0 (1.0, 1.0) 0.109 | 1.0 (1.0, 1.0) <0.001 | 1.0 (1.0, 1.0) <0.001 | 1.0 (1.0, 1.0) 0.017 | 1.0 (1.0, 1.0) <0.001 | 1.0 (1.0, 1.0) <0.001 |
| > Inflection point | 1.0 (0.9, 1.0) 0.036 | 1.0 (1.0, 1.0) 0.808 | 1.0 (1.0, 1.0) 0.168 | 1.0 (1.0, 1.0) 0.986 | 1.0 (1.0, 1.0) 0.535 | 1.0 (1.0, 1.0) 0.566 |
| P for log likelyhood ratio | 0.123 | 0.004 | 0.022 | 0.220 | <0.001 | <0.001 |

**Supplemental table 27: The results of subgroup analyses using chronic pulmonary disease as stratified variables**

|  | Creatinine clearance (CG), OR, 95%CI, P value | | | Creatinine clearance (MDRD),  OR, 95%CI, P value | | |
| --- | --- | --- | --- | --- | --- | --- |
| Chronic pulmonary disease | 0 | 1 | Total | 0 | 1 | Total |
| Fitting model using standard binary logistic regression model | 1.0 (1.0, 1.0) <0.001 | 1.0 (1.0, 1.0) 0.383 | 1.0 (1.0, 1.0) <0.001 | 1.0 (1.0, 1.0) <0.001 | 1.0 (1.0, 1.0) 0.637 | 1.0 (1.0, 1.0) <0.001 |
| Fitting model using two-piecewise logistic regression model |  |  |  |  |  |  |
| Inflection point | 78.8 | 124.5 | 78.3 | 84.2 | 55.7 | 73.5 |
| < Inflection point | 1.0 (1.0, 1.0) <0.001 | 1.0 (1.0, 1.0) 0.589 | 1.0 (1.0, 1.0) <0.001 | 1.0 (1.0, 1.0) <0.001 | 1.0 (0.9, 1.0) 0.096 | 1.0 (1.0, 1.0) <0.001 |
| > Inflection point | 1.0 (1.0, 1.0) 0.143 | 1.0 (0.8, 1.1) 0.505 | 1.0 (1.0, 1.0) 0.168 | 1.0 (1.0, 1.0) 0.336 | 1.0 (1.0, 1.0) 0.395 | 1.0 (1.0, 1.0) 0.566 |
| P for log likelyhood ratio | 0.025 | 0.522 | 0.022 | 0.001 | 0.111 | <0.001 |

**Supplemental table 28: The results of subgroup analyses using beta blocker as stratified variables**

|  | Creatinine clearance (CG), OR, 95%CI, P value | | | Creatinine clearance (MDRD),  OR, 95%CI, P value | | |
| --- | --- | --- | --- | --- | --- | --- |
| Beta blocker | 0 | 1 | Total | 0 | 1 | Total |
| Fitting model using standard binary logistic regression model | 1.0 (1.0, 1.0) 0.003 | 1.0 (1.0, 1.0) <0.001 | 1.0 (1.0, 1.0) <0.001 | 1.0 (1.0, 1.0) 0.011 | 1.0 (1.0, 1.0) <0.001 | 1.0 (1.0, 1.0) <0.001 |
| Fitting model using two-piecewise logistic regression model |  |  |  |  |  |  |
| Inflection point | 96.5 | 69.1 | 78.3 | 73.7 | 75 | 73.5 |
| < Inflection point | 1.0 (1.0, 1.0) 0.001 | 1.0 (1.0, 1.0) <0.001 | 1.0 (1.0, 1.0) <0.001 | 1.0 (1.0, 1.0) 0.005 | 1.0 (1.0, 1.0) <0.001 | 1.0 (1.0, 1.0) <0.001 |
| > Inflection point | 1.0 (1.0, 1.0) 0.964 | 1.0 (1.0, 1.0) 0.161 | 1.0 (1.0, 1.0) 0.168 | 1.0 (1.0, 1.0) 0.944 | 1.0 (1.0, 1.0) 0.269 | 1.0 (1.0, 1.0) 0.566 |
| P for log likelyhood ratio | 0.211 | 0.012 | 0.022 | 0.144 | <0.001 | <0.001 |

**Supplemental table 29: The results of subgroup analyses using statin as stratified variables**

|  | Creatinine clearance (CG), OR, 95%CI, P value | | | Creatinine clearance (MDRD),  OR, 95%CI, P value | | |
| --- | --- | --- | --- | --- | --- | --- |
| Statin | 0 | 1 | Total | 0 | 1 | Total |
| Fitting model using standard binary logistic regression model | 1.0 (1.0, 1.0) <0.001 | 1.0 (1.0, 1.0) <0.001 | 1.0 (1.0, 1.0) <0.001 | 1.0 (1.0, 1.0) <0.001 | 1.0 (1.0, 1.0) 0.003 | 1.0 (1.0, 1.0) <0.001 |
| Fitting model using two-piecewise logistic regression model |  |  |  |  |  |  |
| Inflection point | 124.2 | 73.2 | 78.3 | 107.2 | 69.5 | 73.5 |
| < Inflection point | 1.0 (1.0, 1.0) <0.001 | 1.0 (1.0, 1.0) <0.001 | 1.0 (1.0, 1.0) <0.001 | 1.0 (1.0, 1.0) <0.001 | 1.0 (1.0, 1.0) <0.001 | 1.0 (1.0, 1.0) <0.001 |
| > Inflection point | 1.0 (0.9, 1.0) 0.201 | 1.0 (1.0, 1.0) 0.788 | 1.0 (1.0, 1.0) 0.168 | 1.0 (1.0, 1.0) 0.093 | 1.0 (1.0, 1.0) 0.419 | 1.0 (1.0, 1.0) 0.566 |
| P for log likelyhood ratio | 0.380 | 0.020 | 0.022 | 0.008 | 0.005 | <0.001 |

**Supplemental table 30: The results of subgroup analyses using calcium channel blockers as stratified variables**

|  | Creatinine clearance (CG), OR, 95%CI, P value | | | Creatinine clearance (MDRD),  OR, 95%CI, P value | | |
| --- | --- | --- | --- | --- | --- | --- |
| Calcium Channel Blockers | 0 | 1 | Total | 0 | 1 | Total |
| Fitting model using standard binary logistic regression model | 1.0 (1.0, 1.0) <0.001 | 1.0 (1.0, 1.0) 0.040 | 1.0 (1.0, 1.0) <0.001 | 1.0 (1.0, 1.0) <0.001 | 1.0 (1.0, 1.0) 0.090 | 1.0 (1.0, 1.0) <0.001 |
| Fitting model using two-piecewise logistic regression model |  |  |  |  |  |  |
| Inflection point | 80 | 66.9 | 78.3 | 72.4 | 78 | 73.5 |
| < Inflection point | 1.0 (1.0, 1.0) <0.001 | 1.0 (1.0, 1.0) <0.001 | 1.0 (1.0, 1.0) <0.001 | 1.0 (1.0, 1.0) <0.001 | 1.0 (1.0, 1.0) <0.001 | 1.0 (1.0, 1.0) <0.001 |
| > Inflection point | 1.0 (1.0, 1.0) 0.065 | 1.0 (1.0, 1.0) 0.277 | 1.0 (1.0, 1.0) 0.168 | 1.0 (1.0, 1.0) 0.836 | 1.0 (1.0, 1.0) 0.023 | 1.0 (1.0, 1.0) 0.566 |
| P for log likelyhood ratio | 0.147 | 0.005 | 0.022 | 0.007 | 0.002 | <0.001 |

**Supplemental table 31: The results of subgroup analyses using angiotensin-converting enzyme inhibitor as stratified variables**

|  | Creatinine clearance (CG), OR, 95%CI, P value | | | Creatinine clearance (MDRD),  OR, 95%CI, P value | | |
| --- | --- | --- | --- | --- | --- | --- |
| Angiotensin converting enzyme inhibitor | 0 | 1 | Total | 0 | 1 | Total |
| Fitting model using standard binary logistic regression model | 1.0 (1.0, 1.0) <0.001 | 1.0 (1.0, 1.0) <0.001 | 1.0 (1.0, 1.0) <0.001 | 1.0 (1.0, 1.0) <0.001 | 1.0 (1.0, 1.0) 0.002 | 1.0 (1.0, 1.0) <0.001 |
| Fitting model using two-piecewise logistic regression model |  |  |  |  |  |  |
| Inflection point | 83.5 | 72.1 | 78.3 | 70.8 | 77.9 | 73.5 |
| < Inflection point | 1.0 (1.0, 1.0) <0.001 | 1.0 (1.0, 1.0) <0.001 | 1.0 (1.0, 1.0) <0.001 | 1.0 (1.0, 1.0) <0.001 | 1.0 (1.0, 1.0) <0.001 | 1.0 (1.0, 1.0) <0.001 |
| > Inflection point | 1.0 (1.0, 1.0) 0.537 | 1.0 (1.0, 1.0) 0.136 | 1.0 (1.0, 1.0) 0.168 | 1.0 (1.0, 1.0) 0.798 | 1.0 (1.0, 1.0) 0.462 | 1.0 (1.0, 1.0) 0.566 |
| P for log likelyhood ratio | 0.080 | 0.114 | 0.022 | 0.005 | 0.015 | <0.001 |

**Supplemental table 32: The results of subgroup analyses using left ventricular ejection fraction as stratified variables**

|  | Creatinine clearance (CG), OR, 95%CI, P value | | | | Creatinine clearance (MDRD),  OR, 95%CI, P value | | | |
| --- | --- | --- | --- | --- | --- | --- | --- | --- |
| Left ventricular ejection fraction, mean (sd), % | <40 | >=40, <50 | >=50 | Total | <40 | >=40, <50 | >=50 | Total |
| Fitting model using standard binary logistic regression model | 1.0 (1.0, 1.0) 0.025 | 1.0 (1.0, 1.0) 0.069 | 1.0 (1.0, 1.0) <0.001 | 1.0 (1.0, 1.0) <0.001 | 1.0 (1.0, 1.0) 0.038 | 1.0 (1.0, 1.0) 0.045 | 1.0 (1.0, 1.0) <0.001 | 1.0 (1.0, 1.0) <0.001 |
| Fitting model using two-piecewise logistic regression model |  |  |  |  |  |  |  |  |
| Inflection point | 71.4 | 28.7 | 73.1 | 78.4 | 34.4 | 31.8 | 74.3 | 73.5 |
| < Inflection point | 1.0 (1.0, 1.0) 0.023 | 1.1 (1.0, 1.2) 0.135 | 1.0 (1.0, 1.0) <0.001 | 1.0 (1.0, 1.0) <0.001 | 1.0 (0.9, 1.0) 0.162 | 1.1 (1.0, 1.2) 0.216 | 1.0 (1.0, 1.0) <0.001 | 1.0 (1.0, 1.0) <0.001 |
| > Inflection point | 1.0 (1.0, 1.0) 0.470 | 1.0 (1.0, 1.0) 0.014 | 1.0 (1.0, 1.0) 0.133 | 1.0 (1.0, 1.0) 0.154 | 1.0 (1.0, 1.0) 0.198 | 1.0 (1.0, 1.0) 0.011 | 1.0 (1.0, 1.0) 0.241 | 1.0 (1.0, 1.0) 0.598 |
| P for log likelyhood ratio | 0.321 | 0.043 | 0.050 | 0.023 | 0.329 | 0.088 | <0.001 | <0.001 |

**Supplemental table 33: The results of subgroup analyses using chronic kidney disease requiring dialysis as stratified variables**

|  | Creatinine clearance (CG), OR, 95%CI, P value | | | Creatinine clearance (MDRD),  OR, 95%CI, P value | | |
| --- | --- | --- | --- | --- | --- | --- |
| Chronic kidney disease requiring dialysis | 0 | 1 | Total | 0 | 1 | Total |
| Fitting model using standard binary logistic regression model | 1.0 (1.0, 1.0) <0.001 | NA | 1.0 (1.0, 1.0) <0.001 | 1.0 (1.0, 1.0) <0.001 | 1.5 (0.0, Inf) 1.000 | 1.0 (1.0, 1.0) <0.001 |
| Fitting model using two-piecewise logistic regression model |  |  |  |  |  |  |
| Inflection point | 73.1 | 0 | 73.1 | 72.8 | 6.5 | 72.8 |
| < Inflection point | 1.0 (1.0, 1.0) <0.001 | NA | 1.0 (1.0, 1.0) <0.001 | 1.0 (1.0, 1.0) <0.001 | inf. (0.0, Inf) 1.000 | 1.0 (1.0, 1.0) <0.001 |
| > Inflection point | 1.0 (1.0, 1.0) 0.103 | NA | 1.0 (1.0, 1.0) 0.084 | 1.0 (1.0, 1.0) 0.575 | 0.0 (0.0, Inf) 1.000 | 1.0 (1.0, 1.0) 0.583 |
| P for log likelyhood ratio | 0.007 | 1.000 | 0.007 | <0.001 | 1.000 | <0.001 |

**Supplemental table 34: The results of subgroup analyses using immunodeficiency as stratified variables**

|  | Creatinine clearance (CG), OR, 95%CI, P value | | | Creatinine clearance (MDRD),  OR, 95%CI, P value | | |
| --- | --- | --- | --- | --- | --- | --- |
| Immunodeficiency | 0 | 1 | Total | 0 | 1 | Total |
| Fitting model using standard binary logistic regression model | 1.0 (1.0, 1.0) <0.001 | 1.1 (0.0, Inf) 1.000 | 1.0 (1.0, 1.0) <0.001 | 1.0 (1.0, 1.0) <0.001 | 1.1 (0.0, Inf) 1.000 | 1.0 (1.0, 1.0) <0.001 |
| Fitting model using two-piecewise logistic regression model |  |  |  |  |  |  |
| Inflection point | 78.3 | 93.4 | 78.3 | 73.5 | 39.2 | 73.5 |
| < Inflection point | 1.0 (1.0, 1.0) <0.001 | 1.0 (0.0, Inf) 1.000 | 1.0 (1.0, 1.0) <0.001 | 1.0 (1.0, 1.0) <0.001 | 3.2 (0.0, Inf) 1.000 | 1.0 (1.0, 1.0) <0.001 |
| > Inflection point | 1.0 (1.0, 1.0) 0.155 | 1.5 (0.0, Inf) 1.000 | 1.0 (1.0, 1.0) 0.168 | 1.0 (1.0, 1.0) 0.497 | 0.9 (0.0, Inf) 1.000 | 1.0 (1.0, 1.0) 0.566 |
| P for log likelyhood ratio | 0.021 | 1.000 | 0.022 | <0.001 | 1.000 | <0.001 |

**Supplemental table 35: The results of subgroup analyses using new york heart association class as stratified variables**

|  | Creatinine clearance (CG), OR, 95%CI, P value | | | | | Creatinine clearance (MDRD),  OR, 95%CI, P value | | | | |
| --- | --- | --- | --- | --- | --- | --- | --- | --- | --- | --- |
| New York Heart Association class1234 | 1 | 2 | 3 | 4 | Total | 1 | 2 | 3 | 4 | Total |
| Fitting model using standard binary logistic regression model | 1.0 (1.0, 1.0) 0.038 | 1.0 (0.9, 1.0) 0.009 | 1.0 (1.0, 1.0) 0.006 | 1.0 (1.0, 1.0) <0.001 | 1.0 (1.0, 1.0) <0.001 | 1.0 (1.0, 1.0) 0.047 | 1.0 (0.9, 1.0) 0.020 | 1.0 (1.0, 1.0) 0.046 | 1.0 (1.0, 1.0) <0.001 | 1.0 (1.0, 1.0) <0.001 |
| Fitting model using two-piecewise logistic regression model |  |  |  |  |  |  |  |  |  |  |
| Inflection point | 56.4 | 98.2 | 92.3 | 124.5 | 78.3 | 90 | 76.7 | 55.9 | 69.6 | 73.5 |
| < Inflection point | 1.0 (0.9, 1.0) 0.002 | 1.0 (0.9, 1.0) 0.135 | 1.0 (1.0, 1.0) <0.001 | 1.0 (1.0, 1.0) <0.001 | 1.0 (1.0, 1.0) <0.001 | 1.0 (1.0, 1.0) 0.006 | 0.9 (0.9, 1.0) 0.018 | 1.0 (1.0, 1.0) 0.041 | 1.0 (1.0, 1.0) <0.001 | 1.0 (1.0, 1.0) <0.001 |
| > Inflection point | 1.0 (1.0, 1.0) 0.921 | 0.0 (0.0, Inf) 0.984 | 1.0 (1.0, 1.0) 0.369 | 0.9 (0.8, 1.0) 0.254 | 1.0 (1.0, 1.0) 0.168 | 1.0 (1.0, 1.0) 0.337 | 1.0 (0.9, 1.0) 0.681 | 1.0 (1.0, 1.0) 0.553 | 1.0 (1.0, 1.0) 0.445 | 1.0 (1.0, 1.0) 0.566 |
| P for log likelyhood ratio | 0.030 | 0.041 | 0.013 | 0.258 | 0.022 | 0.075 | 0.317 | 0.194 | 0.002 | <0.001 |

**Supplemental figure 1:Smooth curve fitting diagram under different adjustment strategies**

Supplemental Figure 1 legends: We showed the nonlinearity between creatinine clearance and outcome. We used different adustment strategies. Adjust I：we adjusted for all covariates presented table 1; Adjust II：Adjust I + cardiac surgery （coronary artery surgery, valve surgery, aortic valve surgery, mitral valve surgery, tricuspid valve surgery, and thoracic aortic surgery

**Supplemental figure 2:Smooth curve fitting diagram using age as stratified variables**


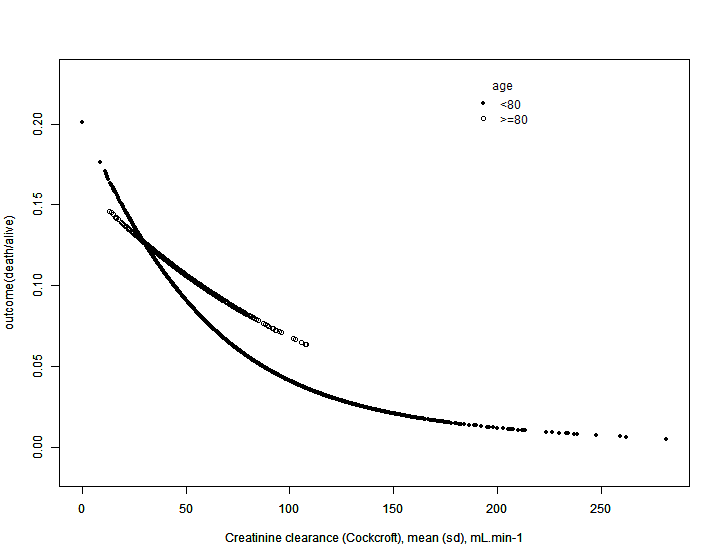

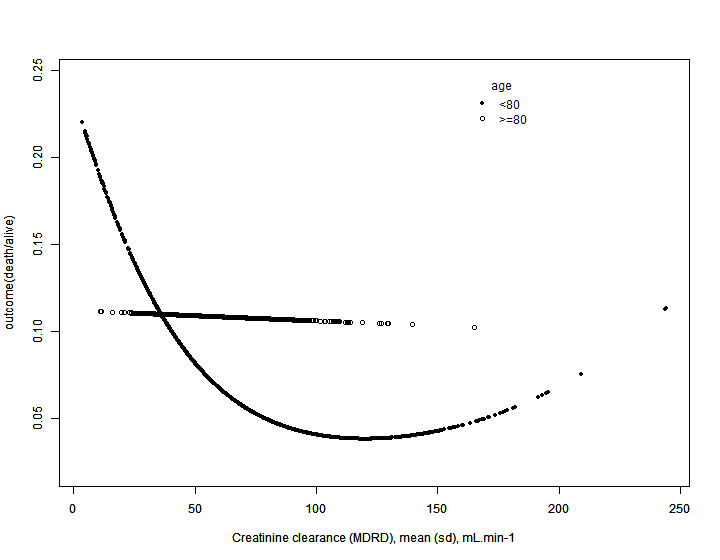


**Supplemental figure 3:Smooth curve fitting diagram using sex as stratified variables**


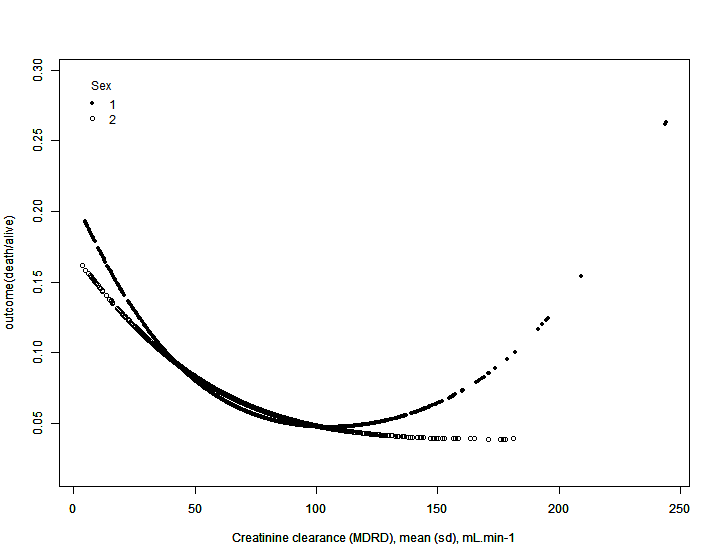

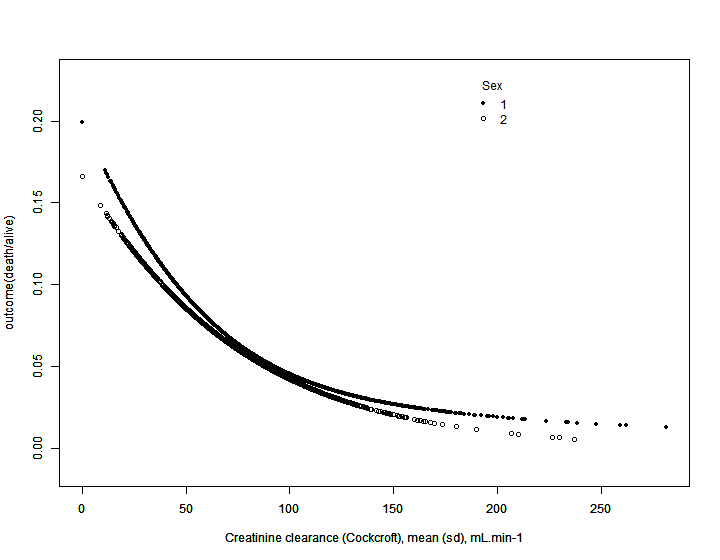


**Supplemental figure 4:Smooth curve fitting diagram using BMI as stratified variables**


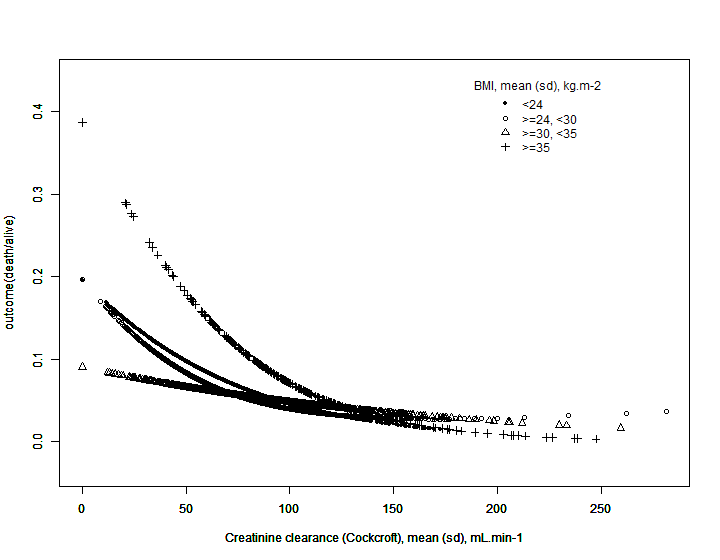

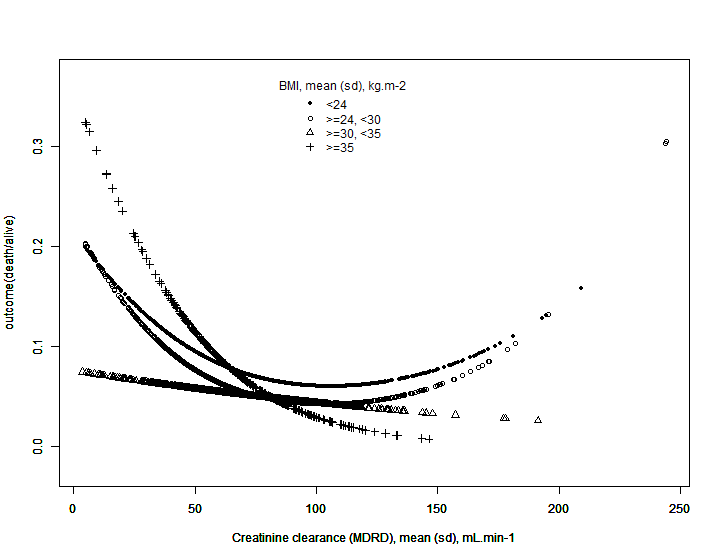


**Supplemental figure 5:Smooth curve fitting diagram using smoking status as stratified variables**


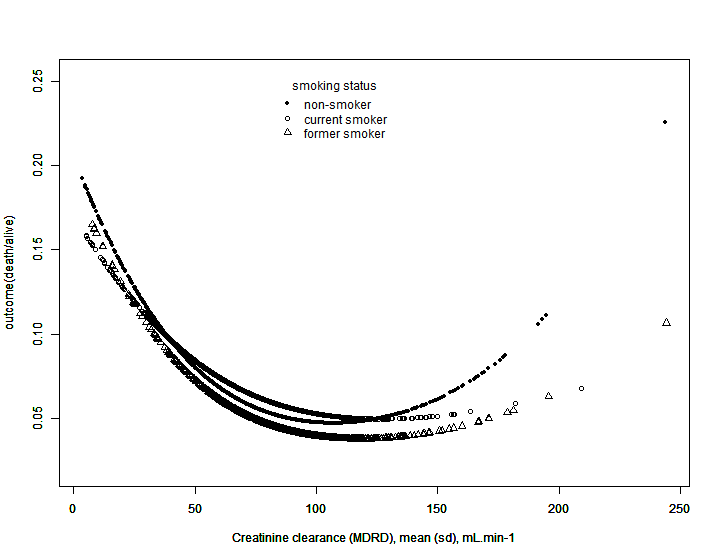

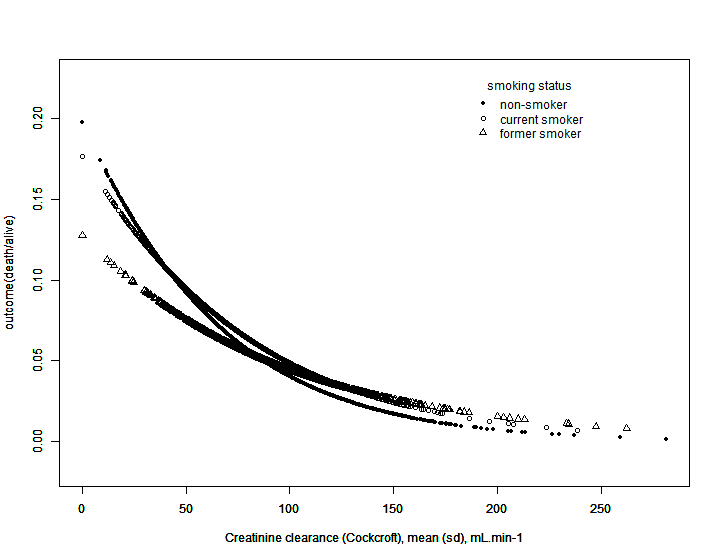


**Supplemental figure 6:Smooth curve fitting diagram using poor mobility as stratified variables**


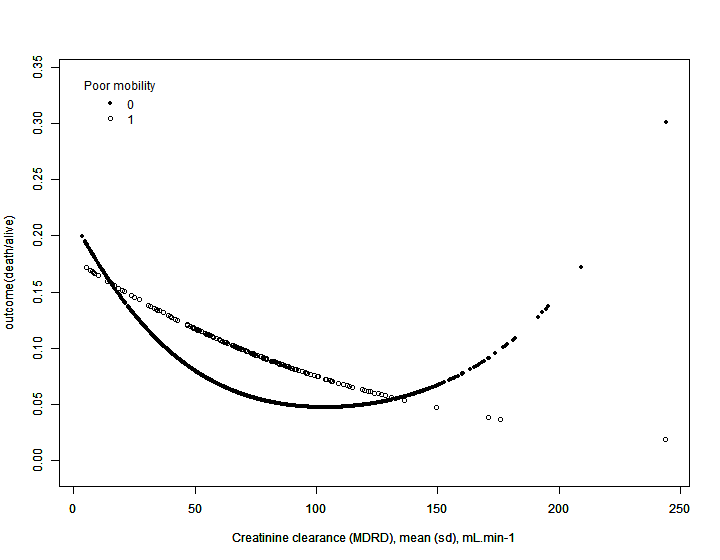

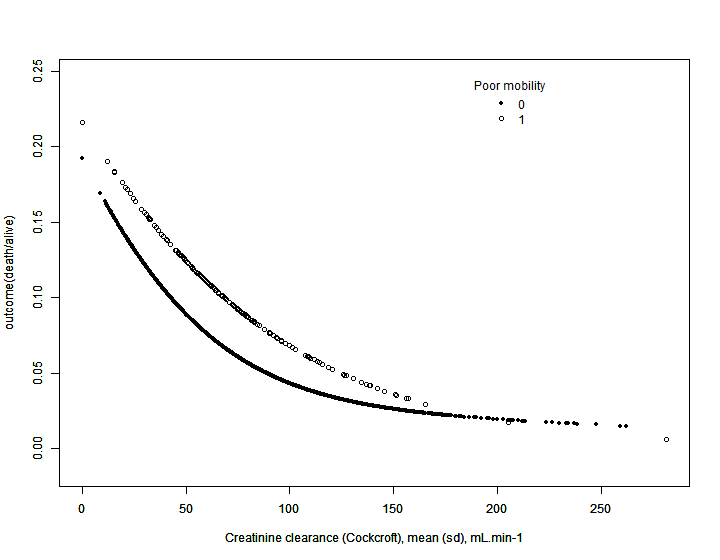


**Supplemental figure 7:Smooth curve fitting diagram using hypertension as stratified variables**


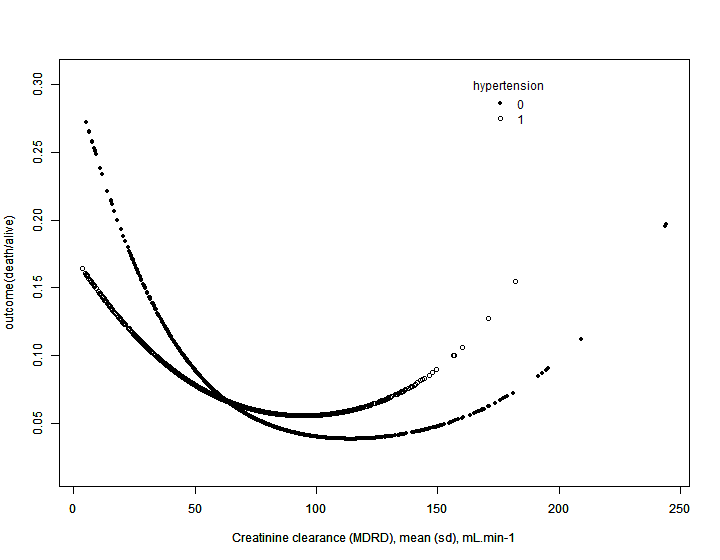

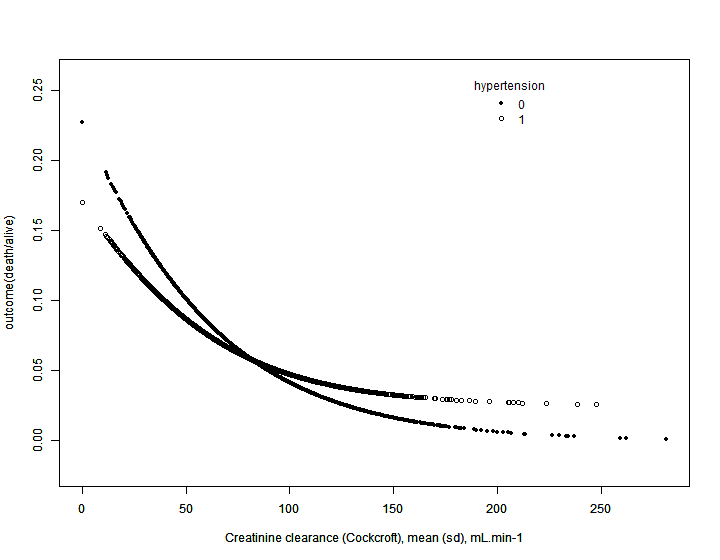


**Supplemental figure 8:Smooth curve fitting diagram using dyslipidemia as stratified variables**


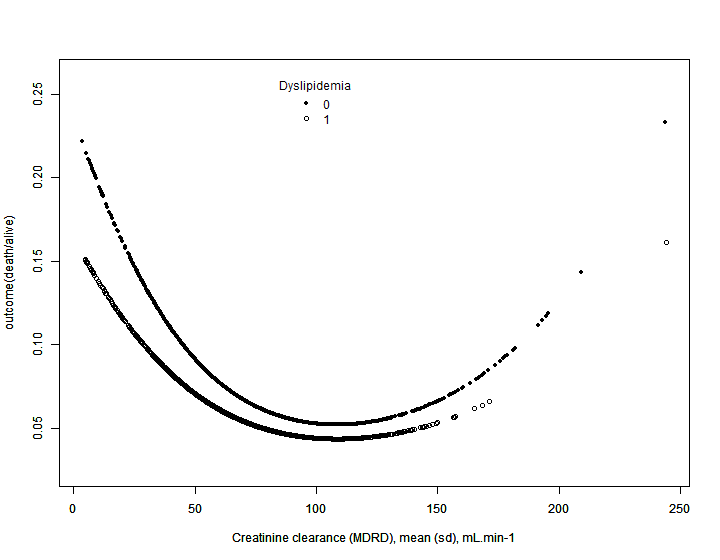

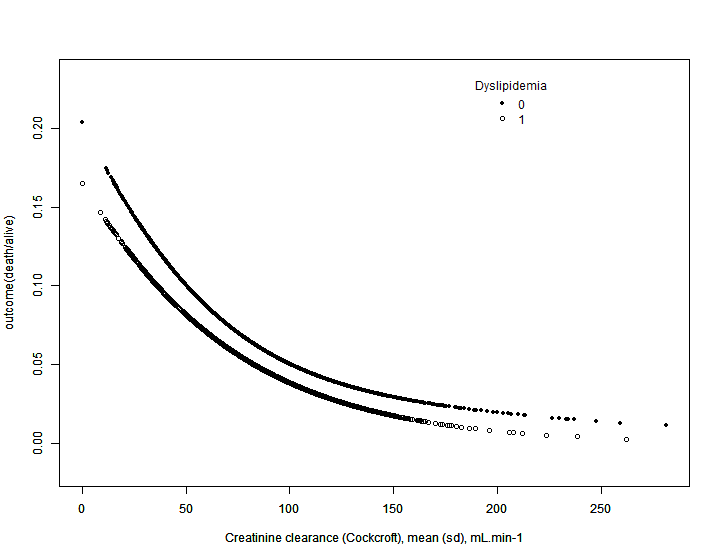


**Supplemental figure 9:Smooth curve fitting diagram using diabetes mellitus as stratified variables**


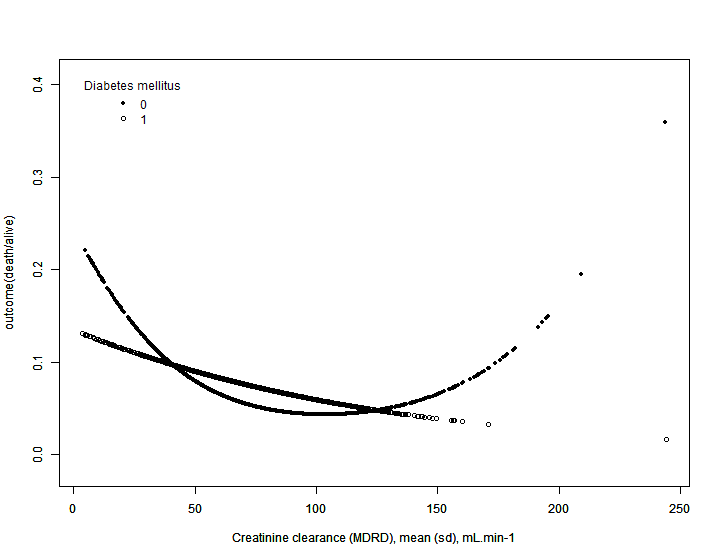

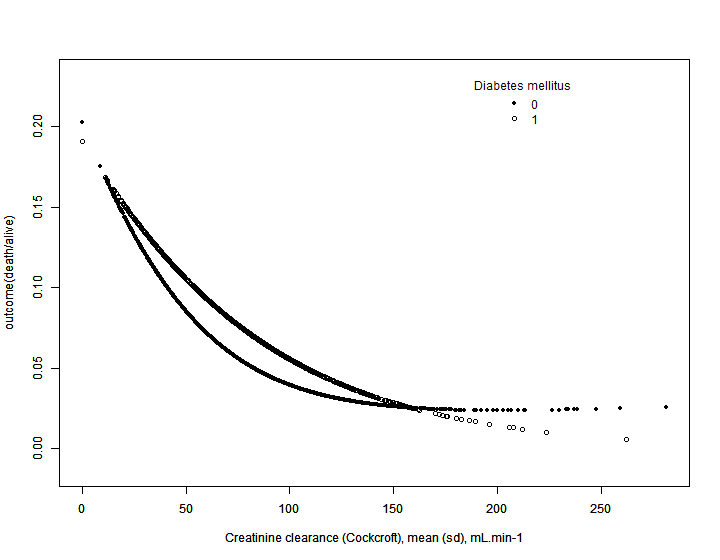


**Supplemental figure 10:Smooth curve fitting diagram using peripheral vascular disease as stratified variables**


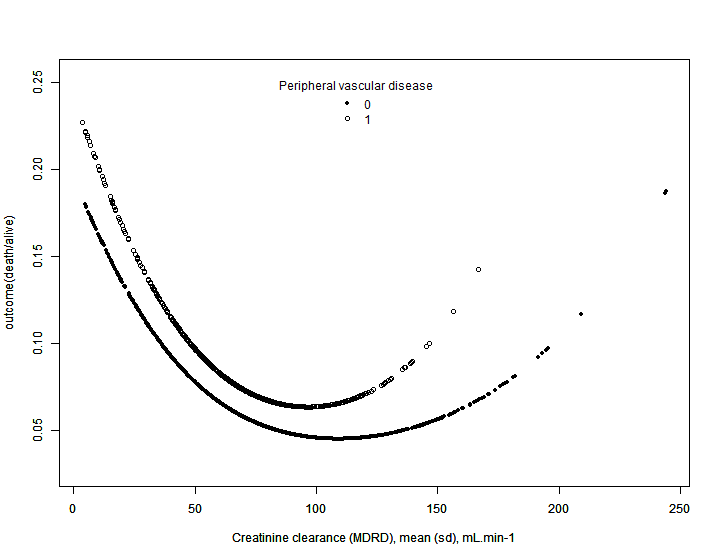

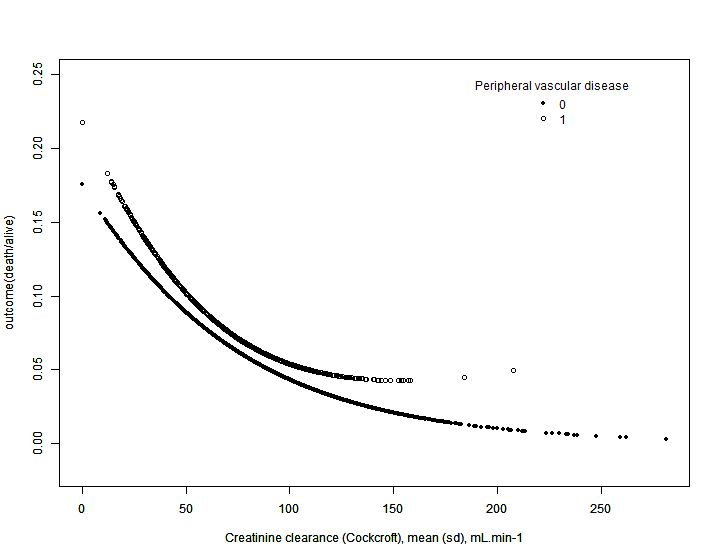


**Supplemental figure 11:Smooth curve fitting diagram using history of thromboembolic event as stratified variables**


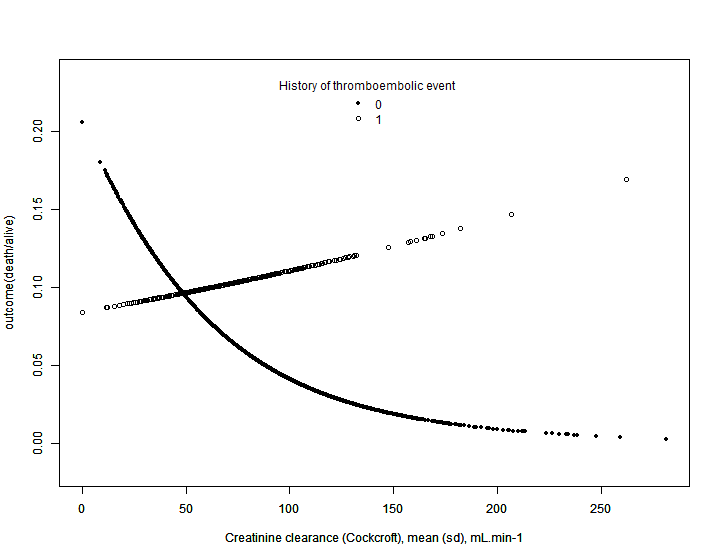

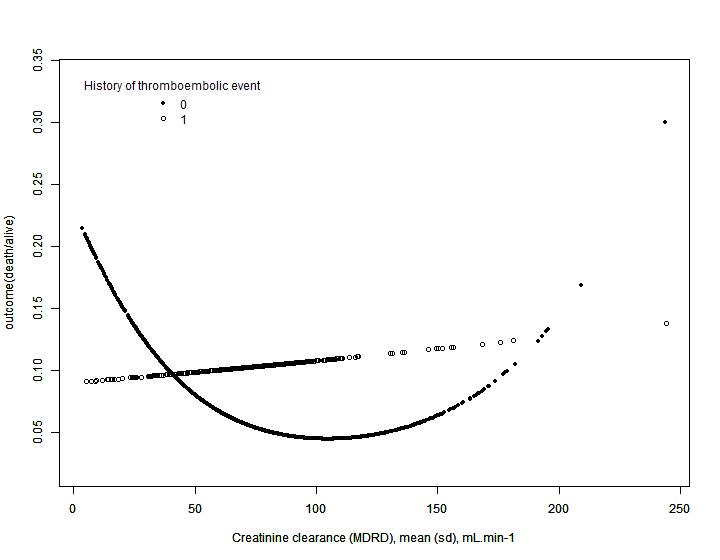


**Supplemental figure 12:Smooth curve fitting diagram using history of coronary artery disease as stratified variables**


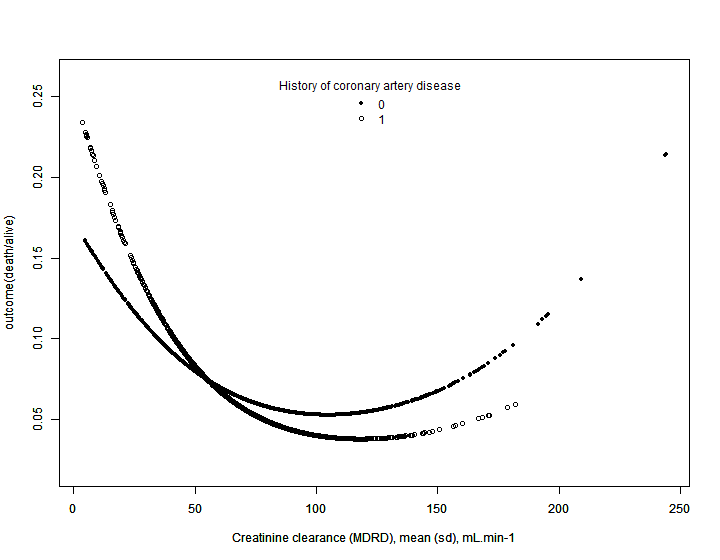

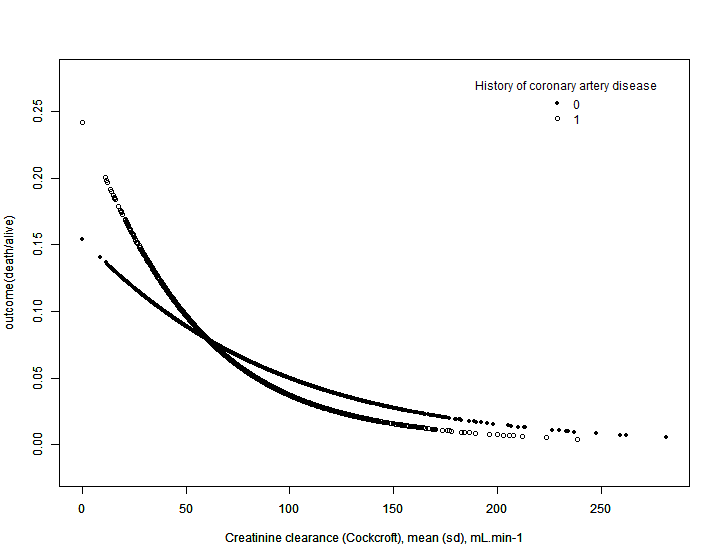


**Supplemental figure 13:Smooth curve fitting diagram using mycardial infarction < 90 days as stratified variables**


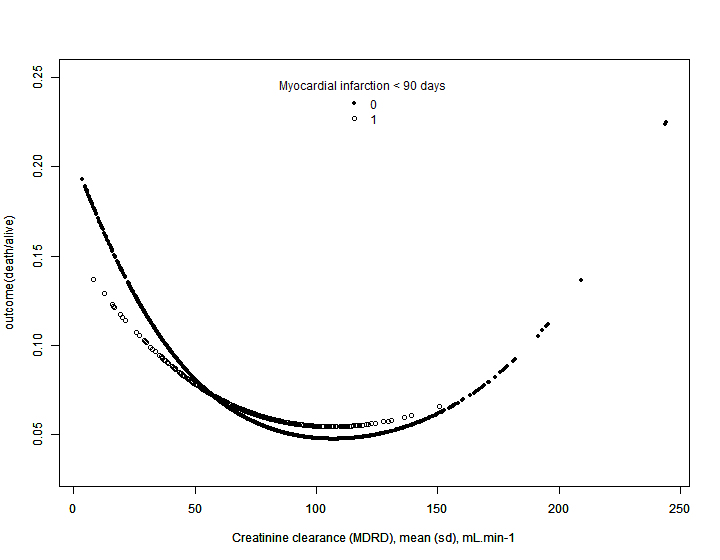

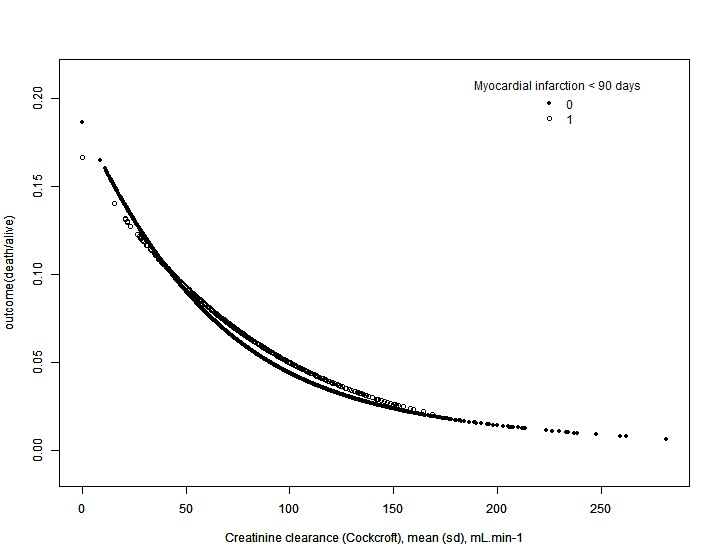


**Supplemental figure 14:Smooth curve fitting diagram using history of cardiac congestive failure as stratified variables**


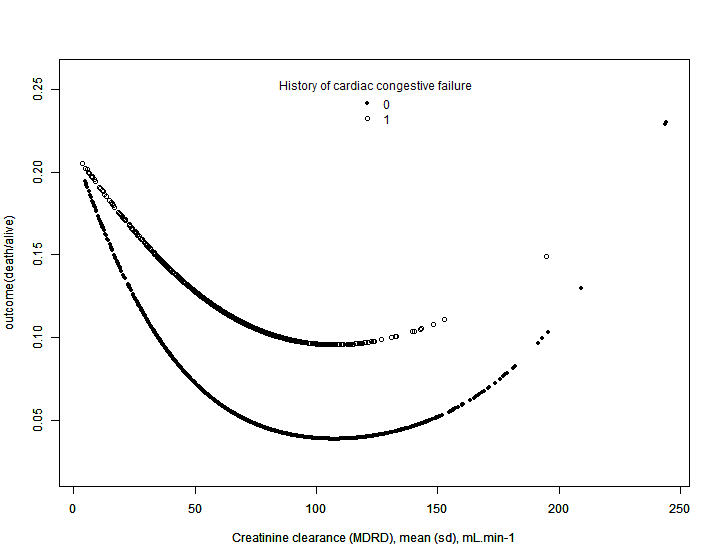

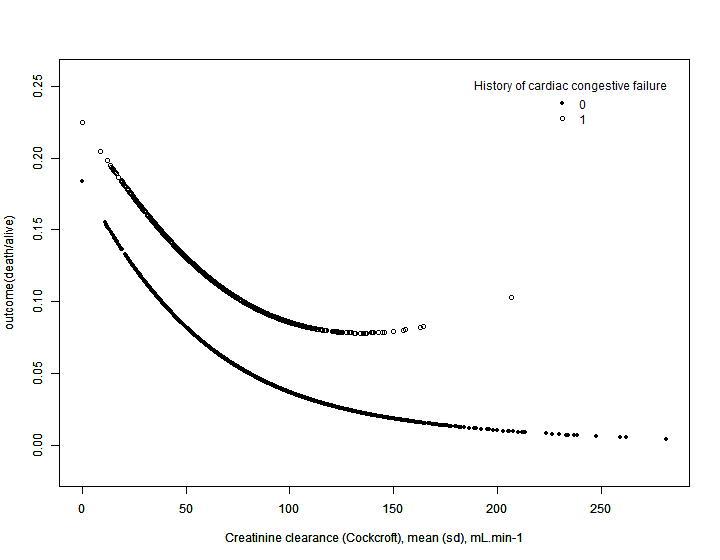


**Supplemental figure 15:Smooth curve fitting diagram using valve disease as stratified variables**


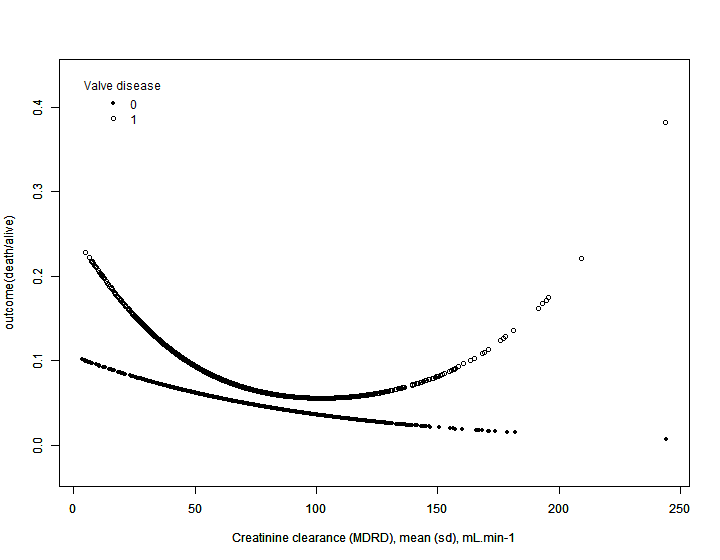

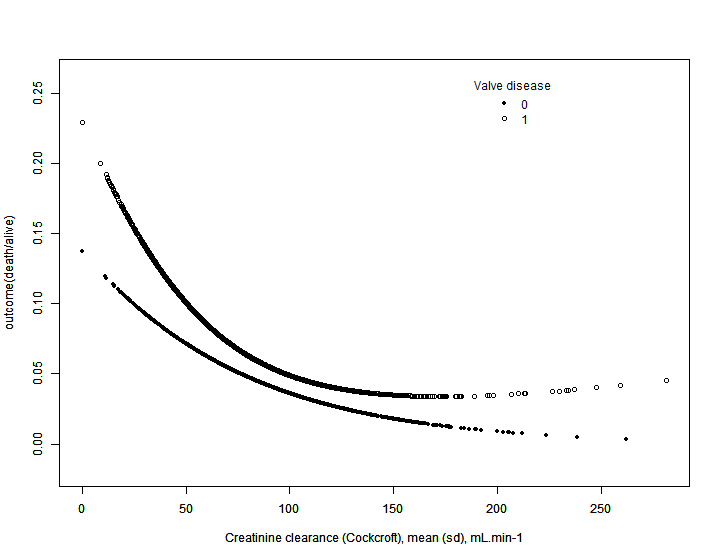


**Supplemental figure 16:Smooth curve fitting diagram using chronic pulmonary disease as stratified variables**


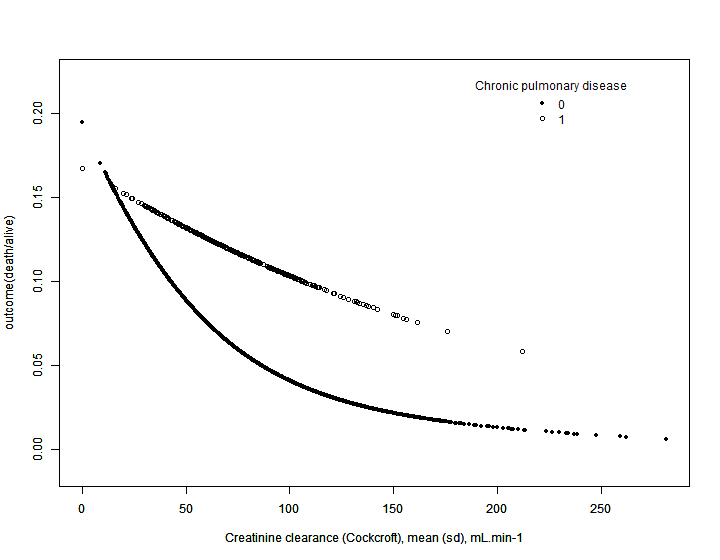

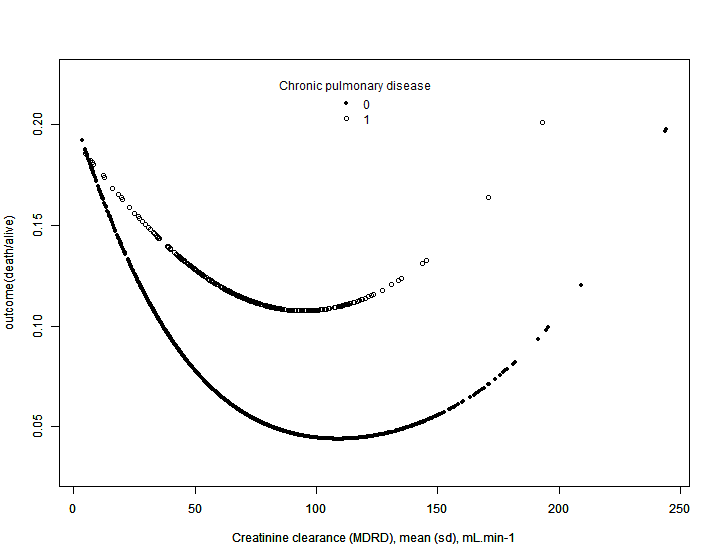


**Supplemental figure 17:Smooth curve fitting diagram using beta blocker as stratified variables**


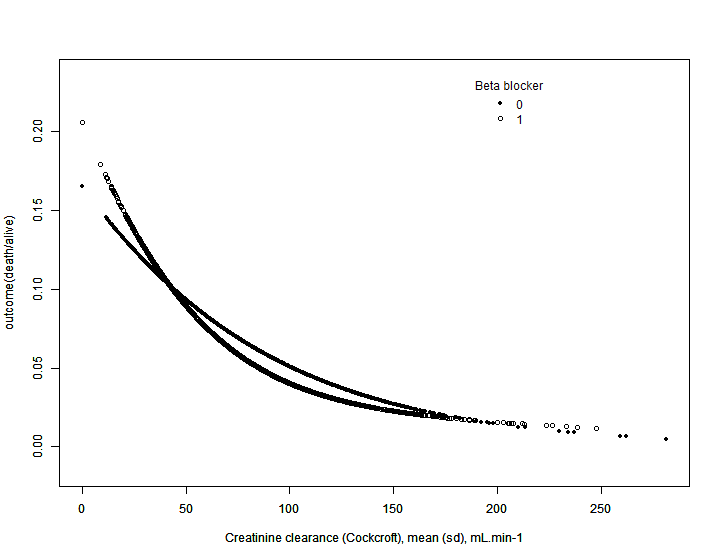

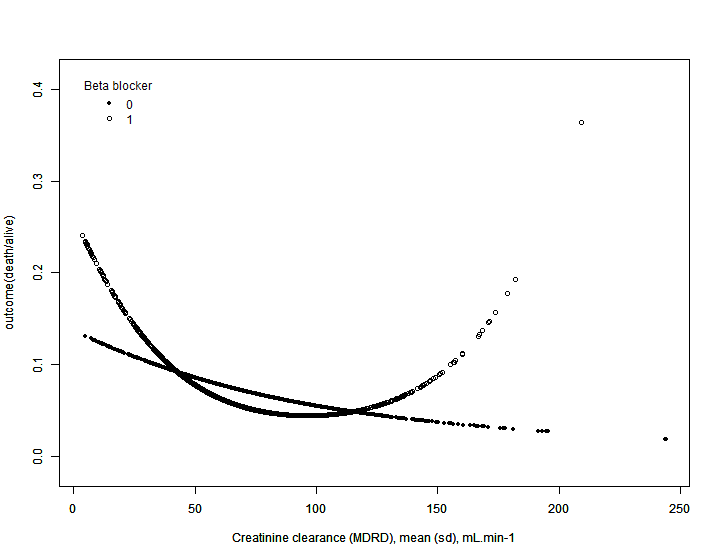


**Supplemental figure 18:Smooth curve fitting diagram using statin as stratified variables**


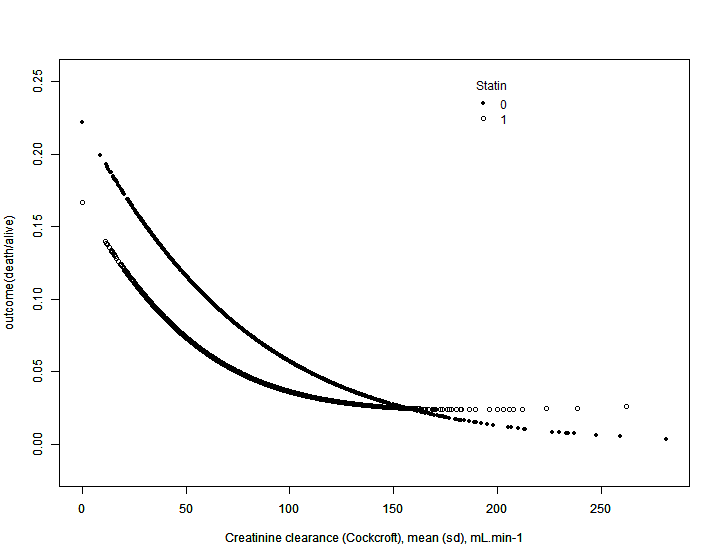

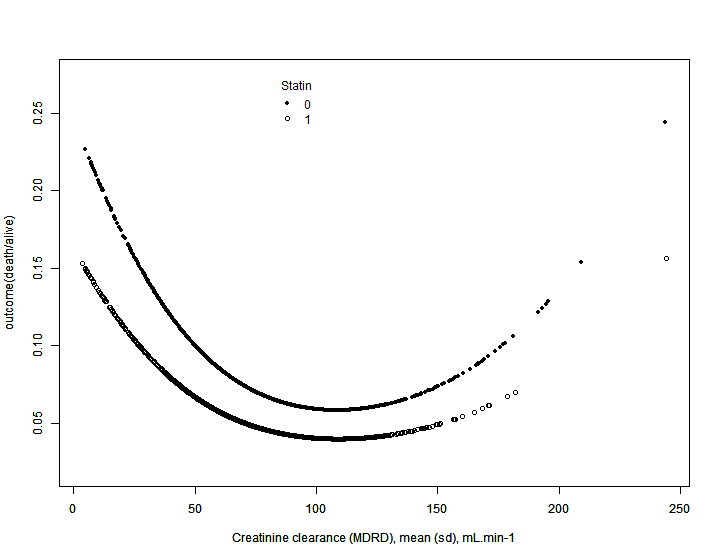


**Supplemental figure 19:Smooth curve fitting diagram using calcium channel blockers as stratified variables**


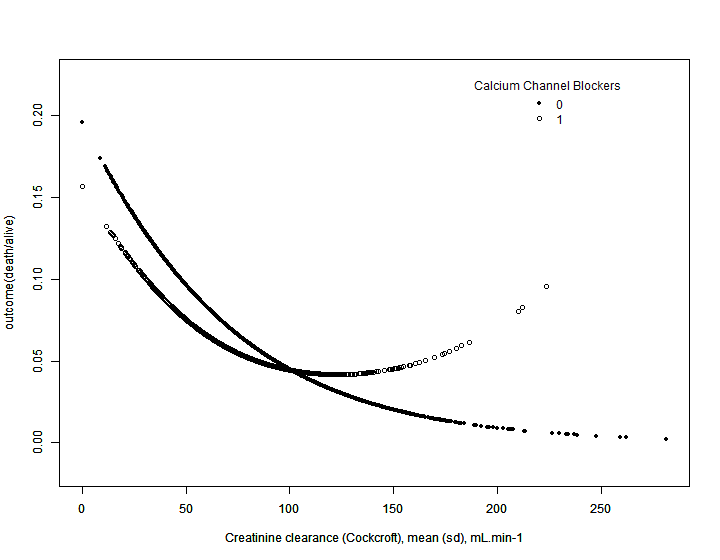

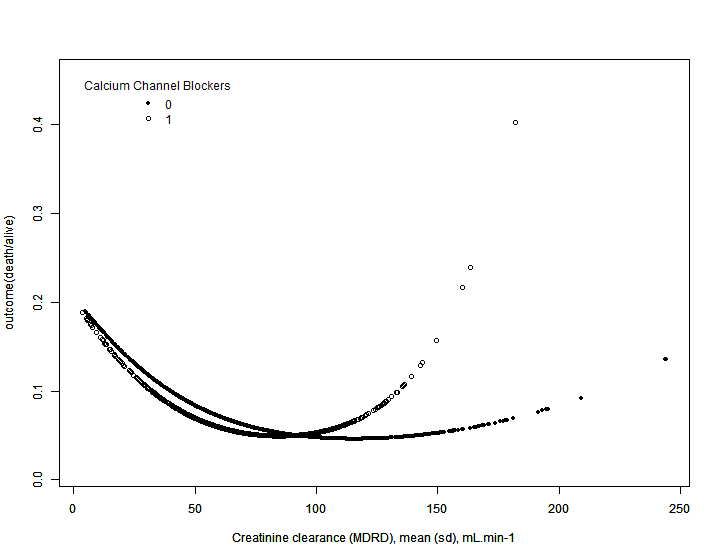


**Supplemental figure 20:Smooth curve fitting diagram using angiotensin-converting enzyme inhibitor as stratified variables**


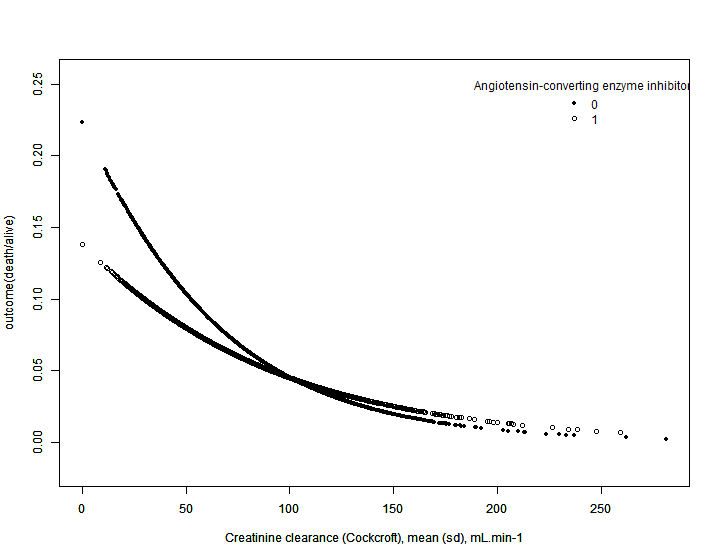

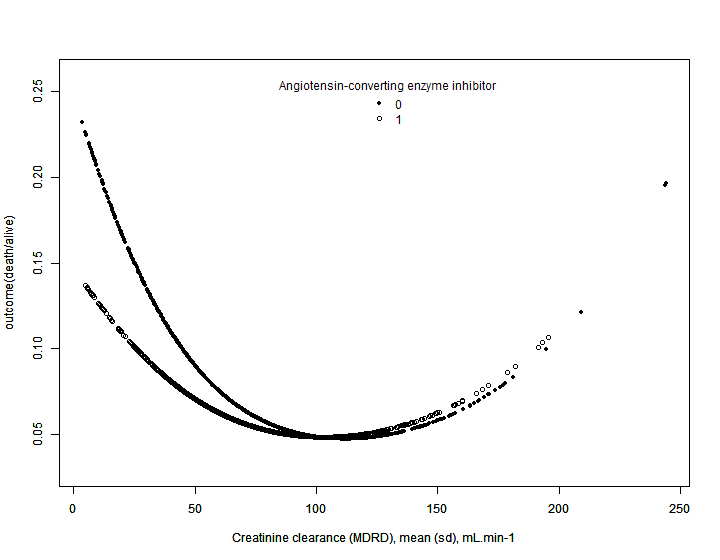


**Supplemental figure 21:Smooth curve fitting diagram using left ventricular ejection fraction as stratified variables**


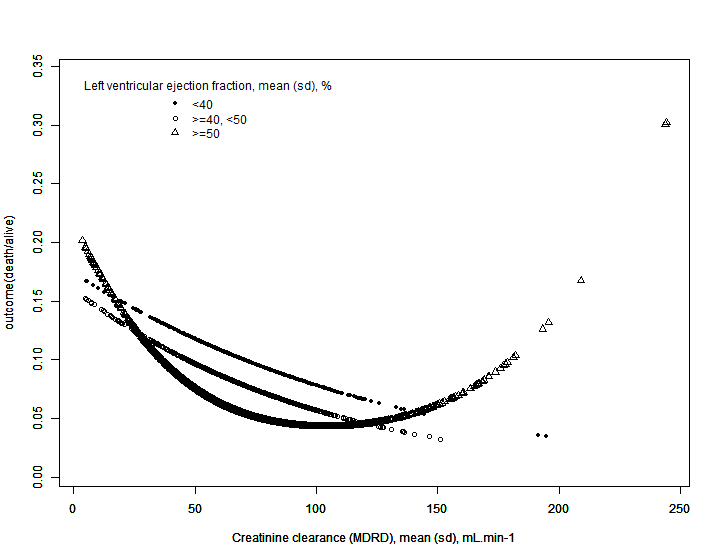

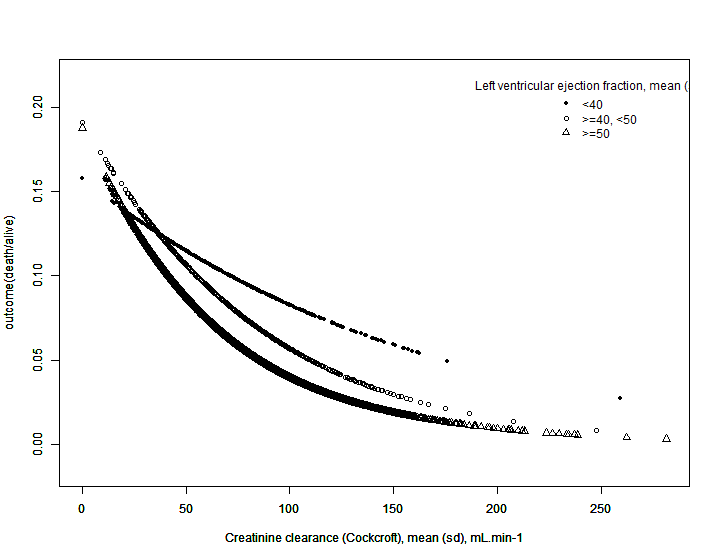


**Supplemental figure 22:Smooth curve fitting diagram using immunodeficiency as stratified variables**


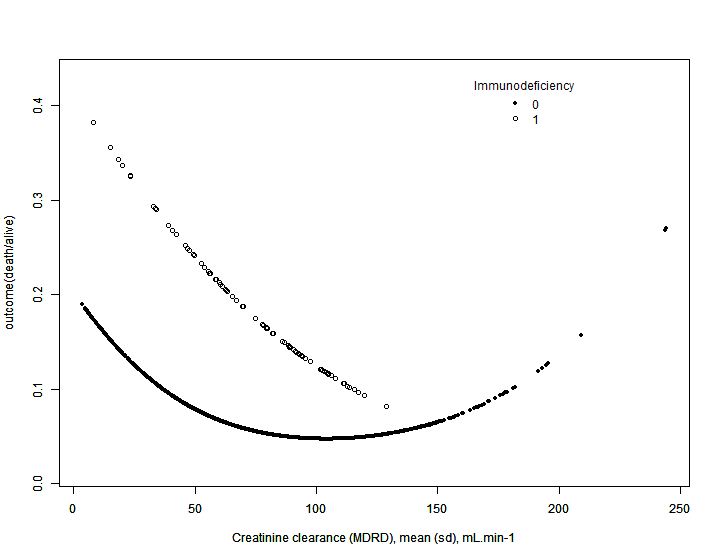

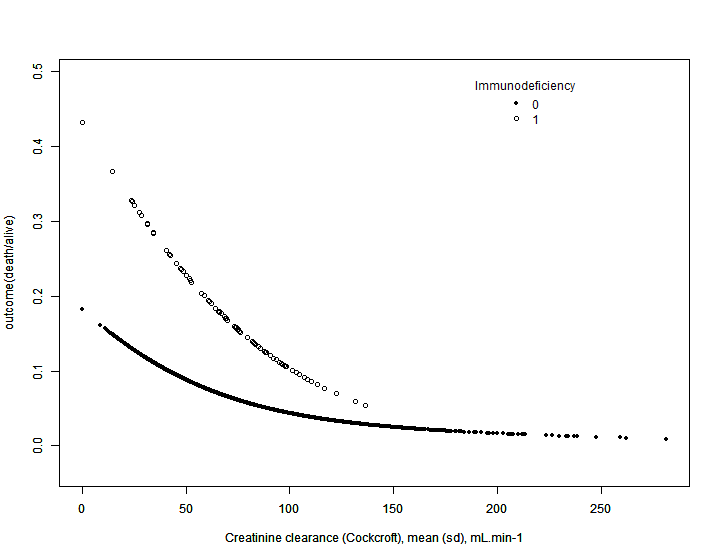


**Supplemental figure 23:Smooth curve fitting diagram using new york heart association class as stratified variables**


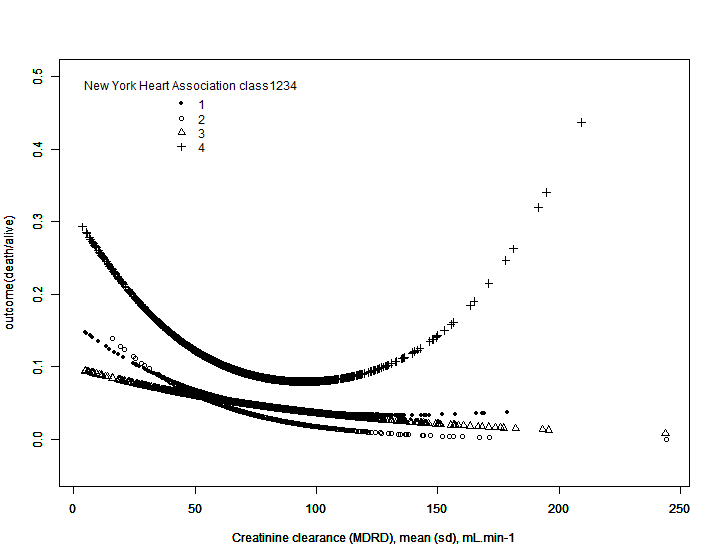

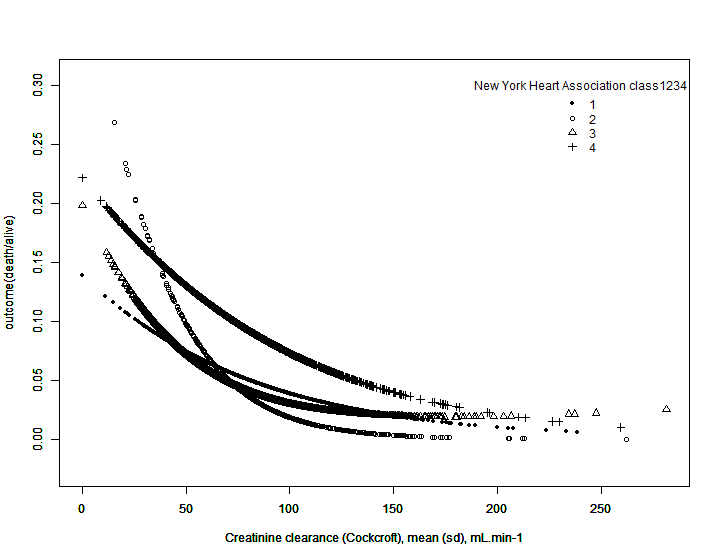

Supplement: Supplementary file 1 [file Data_Sheet_1.docx]
